# Monomorphic genotypes within a generalist lineage of *Campylobacter jejuni* show signs of global dispersion

**DOI:** 10.1099/mgen.0.000088

**Published:** 2016-10-21

**Authors:** Ann-Katrin Llarena, Ji Zhang, Minna Vehkala, Niko Välimäki, Marjaana Hakkinen, Marja-Liisa Hänninen, Mati Roasto, Mihkel Mäesaar, Eduardo Taboada, Dillon Barker, Giuliano Garofolo, Cesare Cammà, Elisabetta Di Giannatale, Jukka Corander, Mirko Rossi

**Affiliations:** ^1^​ Department of Food Hygiene and Environmental Health, Faculty of Veterinary Medicine, University of Helsinki, Helsinki, Finland; ^2^​ Institute of Veterinary, Animal & Biomedical Sciences, College of Sciences, Massey University, Palmerstone North, New Zealand; ^3^​ Department of Mathematics and Statistics, Faculty of Science, University of Helsinki, Helsinki, Finland; ^4^​ Department of Medical and Clinical Genetics, Genome-Scale Biology Research Program, University of Helsinki, Helsinki, Finland; ^5^​ Food and Feed Microbiology Research Unit, Research and Laboratory Department, Finnish Food Safety Authority Evira, Helsinki, Finland; ^6^​ Department of Food Hygiene, Institute of Veterinary Medicine and Animal Sciences, Estonian University of Life Sciences, Tartu, Estonia; ^7^​ Veterinary and Food Laboratory, VFL, Tartu, Estonia; ^8^​ National Microbiology Laboratory, Public Health Agency of Canada, c/o Animal Diseases Research Institute, Lethbridge, Canada; ^9^​ National Reference Laboratory for Campylobacter, Istituto Zooprofilattico Sperimentale dell'Abruzzo e del Molise ‘G. Caporale’, Teramo, Italy; ^10^​ Institute of Basic Medical Sciences, Department of Biostatistics, Faculty of Medicine, University of Oslo, Oslo, Norway

**Keywords:** *Campylobacter jejuni*, genomic epidemiology, monomorphic clones, whole-genome sequencing

## Abstract

The decreased costs of genome sequencing have increased the capability to apply whole-genome sequencing to epidemiological surveillance of zoonotic *Campylobacter jejuni.* However, knowledge of the genetic diversity of this bacteria is vital for inferring relatedness between epidemiologically linked isolates and a necessary prerequisite for correct application of this methodology. To address this issue in *C. jejuni* we investigated the spatial and temporal signals in the genomes of a major clonal complex and generalist lineage, ST-45 CC, by analysing the population structure and genealogy as well as applying genome-wide association analysis of 340 isolates from across Europe collected over a wide time range. The occurrence and strength of the geographical signal varied between sublineages and followed the clonal frame when present, while no evidence of a temporal signal was found. Certain sublineages of ST-45 formed discrete and genetically isolated clades containing isolates with extremely similar genomes regardless of time and location of sampling. Based on a separate data set, these monomorphic genotypes represent successful *C. jejuni* clones, possibly spread around the globe by rapid animal (migrating birds), food or human movement. In addition, we observed an incongruence between the genealogy of the strains and multilocus sequence typing (MLST), challenging the existing clonal complex definition and the use of whole-genome gene-by-gene hierarchical nomenclature schemes for *C. jejuni*.

## Data Summary

1. The raw reads of the isolates sequenced in this study is available under the ENA project PRJEB15115 http://www.ebi.ac.uk/ena/data/view/PRJEB15115.
2. The assembled genomes of strains analysed in this study are available on the PubMLST database (http://pubmlst.org/) under id-numbers presented in Table S1 (available in the online Supplementary Material).
3. Core genome alignments, BEAST analyses, GWAS results, tree files and scripts used in this study are freely available for download at https://github.com/mirossilab/Publications-Data-Scripts/tree/master/ST45-CC.

## Impact Statement

With the high discriminatory power offered by whole-genome sequencing, the spatio-temporal microevolution of the common generalist lineage ST-45 clonal complex of *Campylobacter jejuni* was investigated. We demonstrate that the phylogeographical signal varies considerably between different populations within the clonal complex. Moreover, we described for the first time, to our knowledge, the existence of successful *C. jejuni* clones exhibiting high genetic stability over time and space. We show a persistence of these monomorphic genotypes in animal hosts and their isolation from human patients over a decade from several countries around the globe. Our findings highlight the difficulty of establishing a common framework for WGS-based epidemiological surveillance of zoonotic *C. jejuni*. This arises from the limited genetic variability of monomorphic genotypes and differences in the strength of phylogeographic signals, which complicate the development of a joint cut-off value for determining epidemiological linkage between *C. jejuni* isolates.

## Introduction

The use of whole-genome sequencing (WGS) in genomic epidemiology is revolutionizing surveillance and outbreak investigations of bacterial threats to public health. WGS has been successfully used, for example, to limit the spread of nosocomial methicillin-resistant *Staphylococcus aureus* (Koser *et al.*, 2012), investigate the origin of the Haiti cholera outbreak (Hendriksen *et al.*, 2011) and search for signals of host adaptation for use in source attribution (Dearlove *et al.*, 2015). WGS is currently used in real-time surveillance of *Listeria monocytogenes* and *Salmonella enteritidis* by the American Centers for Disease Control and Prevention and the US Food and Drug Administration (http://www.fda.gov/Food) and similar approaches for *E. coli*, *Campylobacter*, *Vibrio* and *Cronobacter*, etc., are expected to come into use in the near future. In addition, both the European Food Safety Authority (EFSA) and the European Centre for Disease Prevention and Control (ECDC) have emphasized the importance of WGS, and advocate the need for transition from classic laboratory methods to WGS in real-time surveillance of infectious diseases (EFSA, 2014; ECDC, 2015).

*Campylobacter jejuni* is the most common cause of bacterial gastroenteritis worldwide, with an increasing number of cases reported in the EU, including Finland (EFSA & ECDC, 2015; Jaakola *et al.*, 2015). As most cases are self-limiting and unreported and since large point-source outbreaks are rare, identification of sources of *C. jeju*ni is difficult (Blaser & Engberg, 2008). As a result, 30 years of intense research on *C. jejuni* and various mitigation strategies have not been able to reduce the health burden of campylobacteriosis. Improved methods to attribute sporadic cases and detect hidden outbreaks are needed, and thus considerable expectations are directed towards WGS in this regard to ultimately prevent and control the *Campylobacter* epidemic. Applications of WGS for public health purposes are dependent on knowledge of the genomic relationships between isolates, both in the context of outbreaks and sporadic cases. Also, knowledge regarding potential genomic changes occurring through a transmission pathway such as the food chain will be essential in source attribution. According to previous studies on the genetic relatedness of *C. jejuni* circulating in outbreaks and clustering in time and space in chickens, genetic diversity varies between multilocus sequence types (STs) and clonal complexes (CCs) (Revez *et al.*, 2014a, b; Kivistö *et al.*, 2014; Kovanen *et al.*, 2016). Such differences between lineages and sublineages complicate the development of a universal nomenclature, and more studies on the genetic diversity within and across lineages are warranted.

ST-45 CC is a generalist lineage having a wide range of host animals (Sheppard *et al.*, 2014) and a rapid host-switch rate that appears to erode signs of host-adaption (Dearlove *et al.*, 2015). Within this CC, the founder ST-45 is very heterogeneous by Penner heat-stable serotyping (Dingle *et al.*, 2001), *flaA* short-variable-region typing (Dingle *et al.*, 2002), comparative genomic hybridization (Taboada *et al.*, 2008), stress response analyses (Habib *et al.*, 2009), lipooligosaccharide locus class distributions (Revez & Hänninen, 2012), and whole-genome MLST (Kovanen *et al.*, 2016). The existence of several possible animal host species, lack of host signals and genetic and phenotypic heterogeneity of this CC complicate the use of WGS in epidemiological investigations and source attribution. Therefore, a robust description of the genomic diversity within the ST-45 CC across time and space, the two main factors relevant in public health surveillance, is essential for better understanding of the genetic relationship between two isolates.

Genetic differences within species due to geography are commonly encountered in prokaryotes, for example *Helicobacter pylori* (Linz *et al.*, 2007). In *C. jejuni,* this phenomenon is reflected in the overrepresentation or exclusiveness of different lineages according to geography, such as ST-474 in New Zealand (Müllner *et al.*, 2010) and ST-677 in Finland (de Haan *et al.*, 2010; Kärenlampi *et al.*, 2007). Although members of ST-45 CC have been isolated worldwide, there is a high relative frequency of this CC among Finnish patients and chickens compared with other countries (de Haan *et al.*, 2010; McCarthy *et al.*, 2012; Llarena *et al.*, 2015a). However, due to the limited resolution of MLST, it is unclear whether the Finnish overrepresentation of this lineage is a consequence of the local expansion of a successful clone with limited geographical distribution. In this regard, WGS analysis and genome-wide association studies (GWAS) have the potential to provide insight on the process of evolution that have favoured one lineage over another.

The degree of genetic diversity over time varies considerably between prokaryotes, which has strong implications for the applicability of WGS in pathogen surveillance. For instance, *Yersinia pestis* has been under strong purifying selection and has been nearly unaltered since the Black plague (Achtman, 2012), while Morelli *et al.* (2010) found at least 124 single-nucleotide variants over a 40 000 nt region accumulated during a decade in *H. pylori*. Neither long-term evolutionary studies nor studies on the evolutionary change over time in a natural population setting are currently available for *C. jejuni.* Wilson *et al.* (2009) proposed an absolute mutation rate for *C. jejuni,* calculated from MLST, of 3.23×10^−5^ substitutions per site per year. This estimate is ten times faster than the one calculated for *H. pylori* (Morelli *et al.*, 2010) and *Pseudomonas aeruginosa* during chronic infections (Smith *et al.*, 2006) and a hundred times faster than estimates for *E. coli* (Reeves *et al.*, 2011). Therefore, without ignoring the limitations of these estimates (Kuo *et al.*, 2009; Morelli *et al.*, 2010), assuming Wilson’s clock rate and hence, consequently the predicted time of divergence of the most recent common ancestor of ST-45 CC [approximately 81 years before present; Dearlove *et al.* (2015)], detectable evolution and separation by time is expected over the course of a decade.

Our main aim was to characterize the variation and diversity in ST-45 CC across time and space. By comparing 340 isolates of British, Finnish and Baltic origin, we searched for spatial and temporal signals in the genomes of ST-45 CC isolates with the ultimate aim of evaluating the applicability of WGS analysis in surveillance and outbreak investigations. We sought to answer the following two questions: how heterogeneous are various ST-45 CC sublineages and how, if at all, do the genomes of this CC vary over time and between countries.

## Methods

### Isolates, genome sequencing and assembly.

In Dataset one, all publicly accessible genomes of ST-45 CC with available metadata (time and location of isolation) and Finnish and Baltic genomes of the ST-45 CC were included, resulting in a collection of 340 genomes of 22 STs of ST-45 CC, of which 13 were considered singleton STs as they accounted for two or fewer isolates, and one genome of the outgroup ST-21. This database consisted of 199 *C. jejuni* genomes acquired from the PubMLST database [http://www.pubmlst.org/; accessed May 2015 (Jolley & Maiden, 2010)] obtained between 2000 and 2012 in the United Kingdom (UK), of which some strains were collected from human campylobacteriosis cases between June 2011 and June 2014 in Oxford, UK, as part of the Oxfordshire sentinel surveillance study (www.pubmlst.org/Oxforshire_sentinel_surveillance). Furthermore, the sequenced genomes of 126 *C. jejuni* ST-45 CC isolates of Finnish origin from 2000 to 2012 were included from earlier studies (Kovanen *et al.*, 2014b; Revez *et al.*, 2014a; Llarena *et al.*, 2015a; Zhang *et al.*, 2015). In addition, 15 *C. jejuni* ST-45 CC isolates of Finnish, Estonian and Lithuanian origin collected between 1999 and 2012 (Kärenlampi *et al.*, 2007; Kovanen *et al.*, 2014a) and one *C. jejuni* ST-21 CC isolate from Estonia collected in 2012 were subjected to WGS using Illumina technology (performed by Institute for Molecular Medicine, Finland, University of Helsinki, Helsinki, Finland). Genome assembly was performed using SPAdes v 3.2 with default settings using the MismatchCorrector function (Bankevich *et al.*, 2012). All genomes included in this study were smaller than 1.8 Mb and assembled with ≤100 contigs.

A second independent dataset (Dataset two), consisting of ST-45 *C. jejuni* isolates of Italian, Canadian and Finnish origin, was selected to test the hypothesis of global dispersion of specific clones (see ‘Population genetics, genealogy reconstruction and pangenome analysis’). Six ST-45 strains isolated from migrating barnacle geese in a previous study (Llarena *et al.*, 2015b) were sequenced as described above. Eight ST-45 isolates from Italian chickens were collected (sample method as in EFSA, 2010) and sequenced through a national surveillance survey (Project code MSAATE0315) on the prevalence of *Campylobacter* in Italian broiler batches in 2015 (http://www.izs.it/IZS/Eccellenza/Centri_nazionali/LNR_-_Campylobacter/Attivita). Library preparation and sequencing was done using Nextera XT library preparation kits and the NextSeq 500 platform, respectively (Illumina) using the v. 2 (300 cycle, 2×150 nt reads) kit. The Canadian isolates were from animal (*n*=40), human clinical (*n*=31), and environmental sources (*n*=11) and collected through a range of *ad-hoc* sampling activities carried out over the years 2004–2011 (Public Health Agency of Canada). Libraries were constructed as described above and sequenced on a MiSeq platform using the v.2 (300 cycle, 2×150 nt reads) or v.3 (600 cycle, 2×300 nt reads) kits. Assembly was done by SPAdes v 3.7.1. with default setting, except for the ‘–careful’ option for mismatch correction (Bankevich *et al.*, 2012).

The isolate collection and metadata are presented in Tables 1 and S1. Solely Dataset one was used in the analyses unless stated otherwise.

**Table 1.** Overview of isolates included in this study See text for details.

**Source**	***n***	**Country**	**Source of genome sequence**
**Dataset one**			
*ST-45 CC*			
Human	144	UK	PubMLST
	36	Finland	Kovanen *et al.* (2014b)
Animal	42	UK	PubMLST
	95	Finland	Llarena *et al.* (2015a), Zhang *et al.* (2015), This study
	3	Estonia	This study
	2	Lithuania	This study
Environment	5	Finland	Kovanen *et al.* (2016)
Unknown	13	UK	PubMLST
*ST-21 CC* (outgroup)			
Animal	1	Estonia	This study
**Dataset two**			
*ST-45*			
Human	31	Canada	Public Health Agency of Canada
Animal	8	Italy	Italian surveillance study
	40	Canada	Public Health Agency of Canada
	6*	Finland	This study
Environment	11	Canada	Public Health Agency of Canada

*Migratory barnacle geese.

### Generation of the core and accessory genome.

Prodigal was used for gene prediction and a multi-fasta file including all translated coding sequences (tCDSs, *n*=581 171) of the *C. jejuni* genomes was assembled. Reciprocal all-versus-all blastp search was performed (threshold *E*≤ 1e^−^^10^) (Altschul *et al.*, 1997) and orthologous groups were determined by orthAgogue and MCL (ignoring *E-*values, percentage match length ≥80 % and inflation value of 1.5) (Enright *et al.*, 2002; Ekseth *et al.*, 2014). The groups of orthologs (GOs) were aligned using muscle and back-translated to nucleotide sequence using Translatorx perl script (Edgar, 2004a, b; Abascal *et al.*, 2010). GOs with a total alignment length less than 300 nt were excluded, resulting in a pangenome of 2664 GOs. Core (1383 GOs, present in ≥99 % of the 340 ST-45 CC isolates) and accessory gene pools (1281 GOs) were extracted and one representative sequence from each of the GOs from the accessory genome was stochastically selected and annotated using Rapid Annotation Server (Aziz *et al.*, 2008). This annotation was transferred to all members of the corresponding GO. The presence of plasmids (pVir and pTet) and integrated elements (CJIE1-5) (Batchelor *et al.*, 2004; Fouts *et al.*, 2005; Hofreuter *et al.*, 2006; Skarp *et al.*, 2015) were inferred using the script coverager_blastx.v4.pl [Data Citation 1]. Genes and genetic structures suggested to be involved in niche adaptation or strain variability (Hofreuter *et al.*, 2008; Sheppard *et al.*, 2013; Vorwerk *et al.*, 2015), including metabolism and antibiotic resistance loci, were selected for further analysis (e.g. association with population structure), while hypothetical and weakly annotated proteins were excluded.

### Population genetics, genealogy reconstruction and pangenome analysis.

Population structure was defined using BAPS 6.0 [the module hierarchical BAPS (hierBAPS)] with default settings (Cheng *et al.*, 2013). The analysis was performed on the part of the concatenated core genome of *C. jejuni* ST-45 CC strains with orthologs in the *C. jejuni* ST-21 strain (1043 GOs). The number of base differences within BAPS clusters was calculated in mega5 (Tamura *et al.*, 2011) based on pairwise base differences averaged over all possible sequence pairs.

For genealogy reconstruction, recombination was identified from the 1043 GOs defined above using BratNextGen with 20 iterations of the HMM estimation algorithm and 100 permutations with 5 % significance threshold (Marttinen *et al.*, 2012). Thereafter, recombination regions were excluded from the alignment using the Perl script exclude_recombination.pl [Data Citation 1], and phylogeny was inferred using a maximum likelihood (ML) tree based on the recombination-free core-genome sequence estimated with RAxML v. 7 under the generalized time-reversible model (GTRGAMMA) with 100 bootstrap replicates (Stamatakis, 2014). Moreover, a pangenome binary matrix was generated for all strains and used as input for an ML tree reconstruction by RAxML, applying the evolutionary model for binary data with 100 bootstrap replicates. Both ML trees were rooted at the split of the outgroup strain. In addition, the isolates, metadata, population structure and the presence or absence of selected genes were colour-coded to both ML trees using iTOL v 3.0. (Ciccarelli *et al.*, 2006).

To test the hypothesis of global dispersion of possible monomorphic genotypes, i.e. BAPS clusters with low level of genetic diversity over time and space, Dataset two with Canadian, Italian and migrating bird isolates (Tables 1 and S1), was compared with one representative of each hierBAPS cluster level 2 from lineage *b* using rapid core genome multi-alignment by Parsnp implemented in the Harvest Suite, ignoring MUMi distance value cut-off (Treangen *et al.*, 2014). The inclusion of a separate dataset analysed with an independent method allowed us to control for undetected biases within the original dataset and analysis framework.

### GWAS and phylogeographic analyses.

To identify genetic signals overrepresented in strains originating from Finland and the Baltic countries (analysed as a group designated ‘Baltic’) or the UK, GWAS was performed using sequence element enrichment analysis as described in Weinert *et al.* (2015), with the minimum and maximum length of k-mers equal to 10 and 100, respectively. The population structure was accounted for by the hierBAPS clusters and the significance threshold used for any single k-mer was 10^−8^. An intensity plot was created by mapping the significant k-mer frequency per 100 nt along the reference genomes of *C. jejuni* M1 (Friis *et al.*, 2010) and 4031 (Revez *et al.*, 2014a) for the isolates of British and Baltic origin, respectively. A cut-off value for significant k-mer frequency of five was used to identify regions of interest. To analyse the distribution of these words in British and Baltic isolates according to population structure, open reading frames of each region were extracted from the reference genome and used to assess the presence of the three regions A, B and C in the dataset using a gene-by-gene approach implemented in Genome Profiler [flag-o, Zhang *et al.* (2015)]. Missing or truncated loci were considered non-present. The association between these regions and the isolates’ geographical origin was assessed using Pearson’s Chi-Square and Fisher exact test within level 1 BAPS clusters (*P*<0.05 considered significant).

### Bayesian evolutionary analysis.

To estimate the genetic variance across time, Finnish isolates collected over 13 years grouping to BAPS clusters 2, 4 and 6 were selected. For each BAPS cluster, the non-recombined core-genome alignment was extracted using Genome Profiler v 2.0 (Zhang *et al.*, 2015) and BratNextGen (Marttinen *et al.*, 2012). Finally, the alignment gaps were removed, resulting in a gap-free core genome alignment of approximately 1.2–1.3 Mb. A dated phylogeny of each BAPS cluster was reconstructed using Bayesian Evolutionary Analysis Sampling Trees (BEAST) v.1.8.0 (Drummond *et al.*, 2012) assuming a constant population size and with prior parameters normally distributed (mean 0.0; sd 1.0). The HKY substitution model with a strict evolutionary clock was used (Hasegawa *et al.*, 1985; Drummond & Bouckaert, 2015), implying a uniform rate of evolution across branches. For the evolutionary clock, a log normal prior (mean −10.0, sd 1.0, initial value 3.0×10^−5^) was used. The Markov Chain Monte Carlo was run with five million iterations, 500 000 burn-in iterations and sampling every subsequent 1000 iterations. The chain was run twice and summarized in one BEAST tree for each BAPS cluster. An exploratory root-to-tip linear regression, using sampling year information for each strain as an independent variable, was performed in TempEst v1.4 on each Bayesian tree (Rambaut *et al.*, 2016). Thereafter, the coalescent time (denoted τ) estimated by BEAST was tentatively calibrated to years as described earlier (Dearlove *et al.*, 2015) using the Wilson absolute mutation rate for *Campylobacter* of 3.23×10^−5^ substitutions per site per year (Wilson *et al.*, 2009).

### Statistics.

The isolates spatial association with population structure was tested by Pearson Chi-Square or Fisher exact-test. Diversity calculations for location and time of sampling in BAPS populations level 1 were done in PAST v 3.11 (Hammer *et al.*, 2001). Correlation between number of STs, years and countries of origin and genetic distance was calculated in SPSS v. 5.1 (International Business Machines). *P*<0.05 were considered significant for these above mentioned statistics.

## Results

### Population structure and core genealogy

**Fig. 1.**
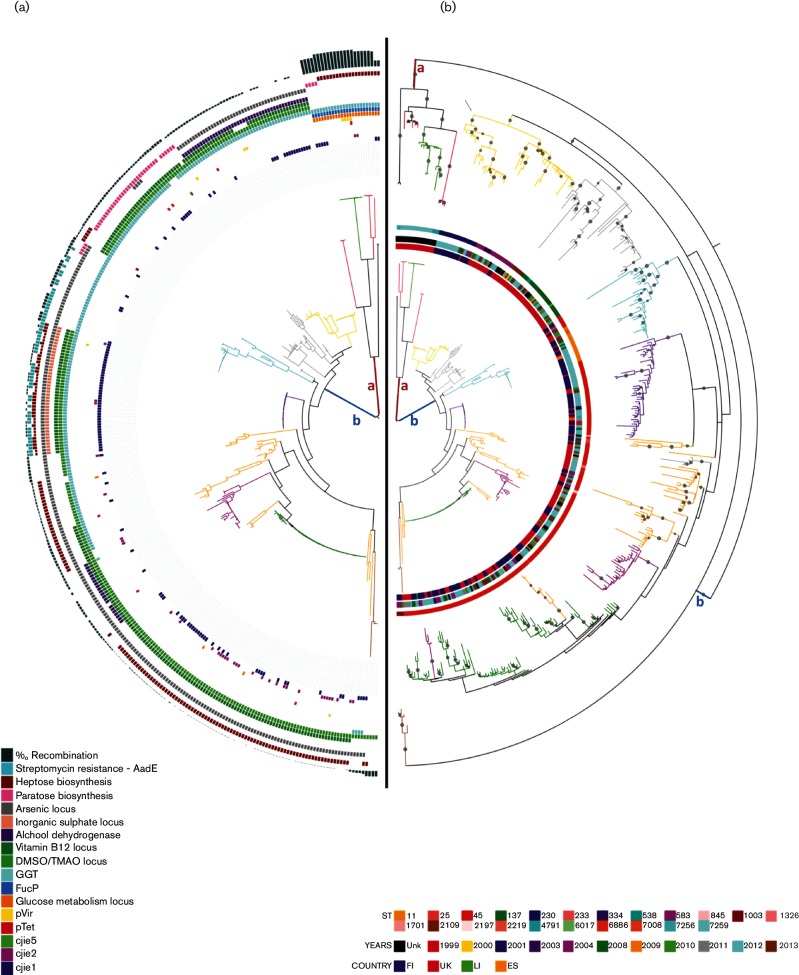
(a) Phylogram of RAxML tree based on non-recombined core genome. Semicircles from outside to inside represent genetic features listed in the legends. Branches are colored according to hierBAPS clustering. The two major clades (*a* and *b*) are indicated. (b) Inner phylogram: RAxML-tree as described for Fig. 1(a). Outer phylogram: RAxML-tree based on a binary matrix of presence–absence of accessory genes. Nodes with bootstrap values >50 % are indicated with a circle. Semicircles from outside to inside: ST, year and country of origin (see legends for indications). Branches are colored according to hierBAPS clustering.

Genealogy divided the isolates into two major clades, *a* and *b*, and the most common STs (ST-45, ST-230, ST-137 and ST-583) were located in clade *b* (Table 2, phylogram Fig. 1a; inner phylogram Fig. 1b; cladogram with bootstrap supporting values ≥80 % in Fig. S1a).

**Table 2.** Population structure of ST-45 CC with associated geographical signal according to clonal frame Geographic association with a country/region is indicated in bold type. EST: Estonia, LIT: Lithuania, FIN: Finland, UK: United Kingdom, BAL: Baltics (FIN, LIT, EST), unk: unknown.

BAPS level 1*	bp. diff.†	*n‡*	STs*	Countries*	Year	Phyly§	Recom||
Clade *a*							
1*	1.7×10^−2^	10	ST-4791, ST-7259	**UK**	unk	Para	0.701
8*	7.4×10^−^^5^	12	ST-7259	**UK**	unk	Mono	0.804
12*	2.7×10^−6^	2	ST-7256	**UK**	unk	Mono	0.262
Clade *b*							
2*	1.5×10^−3^	46	ST-230 **(FIN)**, ST-334, ST-583 **(UK)**	FIN, UK	2004-2013	Mono	0.059
4	2.4×10^−5^	42	ST-45	EST, FIN, UK	1999-2013	Mono	0.042
5	1.0×10^−5^	26	ST-45	LIT, FIN, UK	2008-2013	Mono	0.075
6	9.2×10^−5^	83	ST-45, ST-7008	LIT, FIN, UK	2000-2013	Mono	0.001
7*	4.4×10^−3^	45	ST-25, ST-45, ST-233, ST-686, ST-845, ST-1326, ST-1701, ST-2197	FIN, **UK**¶	2004-2013	Poly	0.054
9*	3.4×10^−3^	42	ST-45, ST-137, ST-538, ST-2109, ST-6017	FIN, **UK**	2000-2013	Poly	0.078
10*	1.6×10^−3^	28	ST-11 **(BAL),** ST-45, ST-2219 **(BAL)**	**BAL**, UK	1999-2013	Mono	0.124
11*	5.1×10^−3^	4	ST-1003	UK, FIN	2004-2010	Mono	0.237

*BAPS populations with evidence of geographical structuring with the country of origin marked in bold.

†Average genetic distance nucleotide in the aligned, non-recombining core genome.

‡Number of strains in each BAPS cluster.

§Phylogeny of the BAPS population according to the core genome ML tree.

||percentage of core genome alignment with signs of recombination originating from outside the dataset.

¶Variable association within the BAPS cluster, as BAPS 22*–24* at the secondary level were associated with UK origin.

Population structure inferred using nested clustering implemented in hierBAPS is summarized in Table 2. First level clustering divided ST-45 CC between 11 populations which split further into 38 subpopulations at the secondary level. The majority of the populations were polychotomously arranged in six monophyletic groups within clade *b* (Figs 1a and S1a, Table S2). Except for the polyphyletic ST-45 and paraphyletic ST-7259, first-level clustering did not divide STs into different populations (Tables 2 and S2). However, with the exception of four STs (ST-230, ST-334, ST-2109 and ST-4791; 37 isolates), polyphyletic or paraphyletic relationships were common at second-level hierBAPS clustering.

The average base differences per nucleotide ranged between 1.7×10^−2^ and 2.7×10^−6^ (Table 2) and a higher genetic diversity (1.7×10^−2^ to 1.5×10^−3^) was observed in BAPS clusters populated by multiple STs (BAPS 1, 2, 7, 9 and 10), also reflected in a positive correlation between genetic distance and diversity of STs within BAPS groups (0.693; *P*=0.018, and 0.722; *P*=0.012). Despite an observed high Simpson index of diversity for location and time of sampling (range =0.79–0.85), low genetic difference was evident for major BAPS groups, especially BAPS 4 (Fig. 1a; blue violet clade), 5 (Fig. 1a; fuchsia clade) and 6 (Fig. 1a; green clade) composed of ST-45.

The geographical signal varied between branches in the core genealogy and BAPS clusters. Evidence of local microevolution was found in clade *b* as the finer level population structure defined by hierBAPS level 2 on some occasions clustered according to geographical origin of the strains (Table 2, in bold type; Fig. 1b; Table S1). For instance, BAPS 9 was British, except for two Finnish isolates which formed the sub-population of BAPS 32* (asterisk denotes second-level hierBAPS). However, due to low genetic distance, the genealogy was unable to separate the populations according to geography for several groups. This was the case for BAPS 4, 5, 6 and 11. The majority of the strains (95.8 %) populating these BAPS groups were ST-45. Since the numbers of isolates are small for the secondary-level clusters, caution related to their interpretation is warranted as the sampling design may have insufficient power to detect signal beyond local microevolution in these cases.

Isolates from a wide range of sampling times were scattered through the ML tree (Fig. 1), with no evidence of monophyletic relationships in terms of time. The population structure could not predict the temporal origin of the isolates in a clear-cut way as all BAPS populations on levels 1 and 2 contained isolates from different years (for BAPS populations above five isolates; Table 2). This lack of genetic structuring in terms of time was reflected in the genetic distance, which was not associated with the number of sampling years covered by the BAPS populations.

### Pangenomic analysis

To search for potential spatial and temporal signals residing in the accessory genome, a ML tree based on the presence and absence of genes was reconstructed. The branch lengths increased considerably with the inclusion of the accessory genome. However, the support for the deepest branches decreased substantially due to, probably, the occurrence of pervasive horizontal gene transfer (bootstrap values ≥80 %, Fig. S1b). The two major clades, *a* and *b*, were still present and the constituents of these two lines remained. However, the polychotomy of clade *b* increased drastically, splitting the six core genealogy-based monophyletic groups into 73 clusters. Nevertheless, the increased resolution residing in the accessory genome revealed low levels of geographical clustering due to spatial local microevolution inside a few populations (i.e. BAPS 6 and 4) which was not apparent in the core genome analysis. No temporal signals were found using a pangenome approach as no grouping according to sampling year was observed.

An association between specific sets of accessory genes and the clonal frame was found across the CC (Fig. 1a. Clade *a* contained strains characterized by the unique combination of genes for glucose and fucose metabolism (*fucP*) and the γ-glutamyl transferase gene (*ggt*). On clade *b*, each monophyletic group was characterized by a specific assortment of genes related to metabolism (Vitamin B5 biosynthesis, *ggt*, alcohol dehydrogenase, arsenic, inorganic sulphate and DMSO/TMAO metabolism), surface structures (heptose and paratose biosynthesis), hypervariable regions (*AadE*, CJIE10–5) and plasmids (pTet and pVir).

### Identification of phylogeographical signal in ST-45 CC isolates using GWAS

**Fig. 2.**
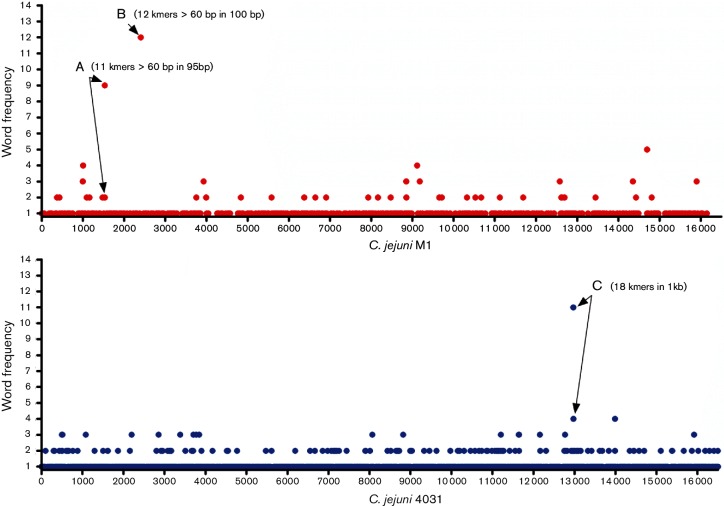
Manhattan plots illustrating significant hits associated with UK (red, upper) or Baltic countries (blue, lower) mapped to *C. jejuni* M1 or *C. jejuni* 4031 genomes, respectively. The dots represent number of words (k-mers), and the regions with higher number of mapped words are indicated with arrows and letters. In the UK Manhattan plot, region A (11 k-mers) mapped to position 153 200–153 295 and region B (12 k-mers) to position 240 500–240 600 on M1. In the lower Baltic plot, region C (18 k-mers) mapped to position 1 276 300–1 277 300 on 4031.

By the use of GWAS, three regions in the accessory genome (marked A, B and C) were identified as overrepresented in strains originating from either the UK or Finland, Estonia and Lithuania, of which the three latter were analysed as a group. The Manhattan plots are presented in Fig. 2, and show that region A and B were enriched in British strains, while region C was overrepresented in the Finnish and Baltic strains. Regions A and B match the C-terminal parts (approximately 100 nt) of two identical methyl-accepting chemotaxis protein paralogs and region C corresponds to a hypothetical protein. As the effects of these signals were uncertain, the distribution of these signals in British and Finnish–Baltic strains according to population structure was assessed. Except for BAPS 4, 5 and 6, in which regions A and B were associated with British isolates, none of the three signals were able to correctly predict geographical origin within populations.

### Searching for temporal signal using Bayesian phylogeny

**Fig. 3.**
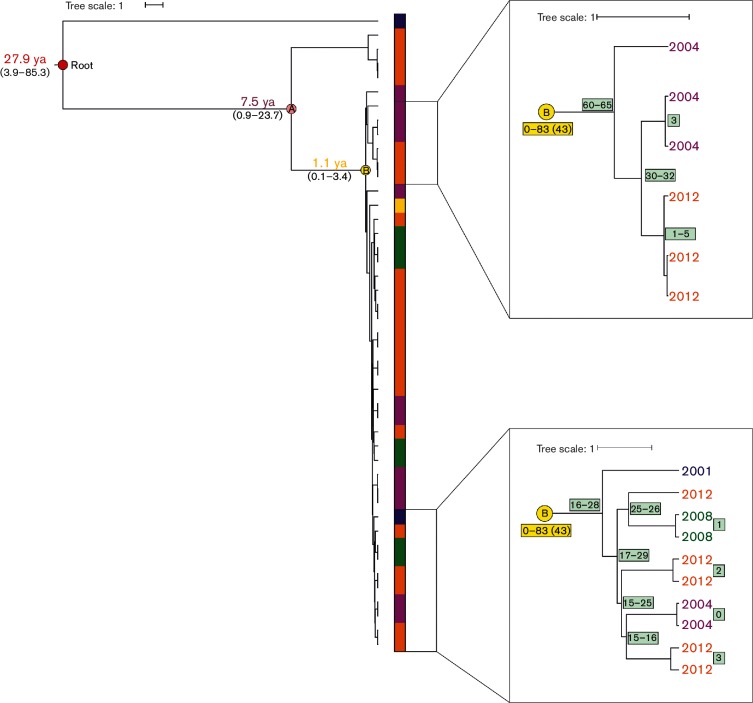
Bayesian phylogenetic tree of Finnish BAPS 6 isolates inferred with BEAST based on non-recombined core genome of approximately 1.2 Mb. Year of isolation is indicated by the color of the bar: yellow: 2000, blue: 2001, purple: 2004, green: 2008, orange: 2012. Two subclusters of BAPS 6 are scaled up and presented as embedded illustrations. Predicted divergence times with confidence intervals given in years before present are given at their respective nodes. Single-nucleotide variant (SNV) numbers in a cluster are given in yellow and light green boxes near the most common recent ancestor of that cluster (when space allows, otherwise located between the involved leafs). Fewer than five SNVs were detected between epidemiologically linked isolates (two from 2008, two from 2012, two from 2004, three from 2012), indicating the usefulness of WGS in outbreak investigations.

The geographical signals in the accessory- and core-genome of ST-45 CC varied between sublineages, implying variation in the occurrence of local microevolution. Such lack of genetic isolation by distance might be due to the monomorphic nature of certain lineages which are more genetically stable over time and space (Achtman, 2012). We therefore investigated the occurrence of a temporal signal using BEAST in Finnish isolates within three populations harbouring low genetic diversity in their core genome and high Simpson index of diversity for year of isolation (range 0.56–0.77): two geographically widespread ST-45 populations (BAPS 4 and 6) and one Finland-associated sublineage [ST-230; BAPS 2(4*)]. Three separate clades were identified in BAPS 6 (Fig. 3), and one of them was populated by strains collected during the whole sampling period. In this clade, the nucleotide diversity in the non-recombined core-genome ranged between 0 and 83, which was similar to the average nucleotide diversity measured between isolates collected during one year (as highlighted in Fig. 3). Using a molecular clock with the mutation rate 3.2×10^–5^nucleotides per year (Wilson *et al.*, 2009), the time of the most common recent ancestor for this cluster was estimated to be 1.1 years before present (node B in Fig. 3), which corresponds poorly with the true sampling points of these strains. In addition, the sampling date had a weak correlation with the root-to-tip distance calculated in TempEst v1.4 (linear regression, R^2^=0.12). Similar results were observed in BAPS 2(4*) and BAPS 4 [BEAST_Analysis_BAPS2.7z and BEAST_Analysis_BAPS4.7z (Data Citation 1)], suggesting that these populations are genetically stable over time, evolving at a much slower rate than previously estimated. This genetic stability observed over time and space indicates that BAPS 4 and BAPS 6 are genetically monomorphic sublineages of ST-45.

### Verifying the presence and global dissemination of monomorphic ST-45 genotypes

**Fig. 4.**
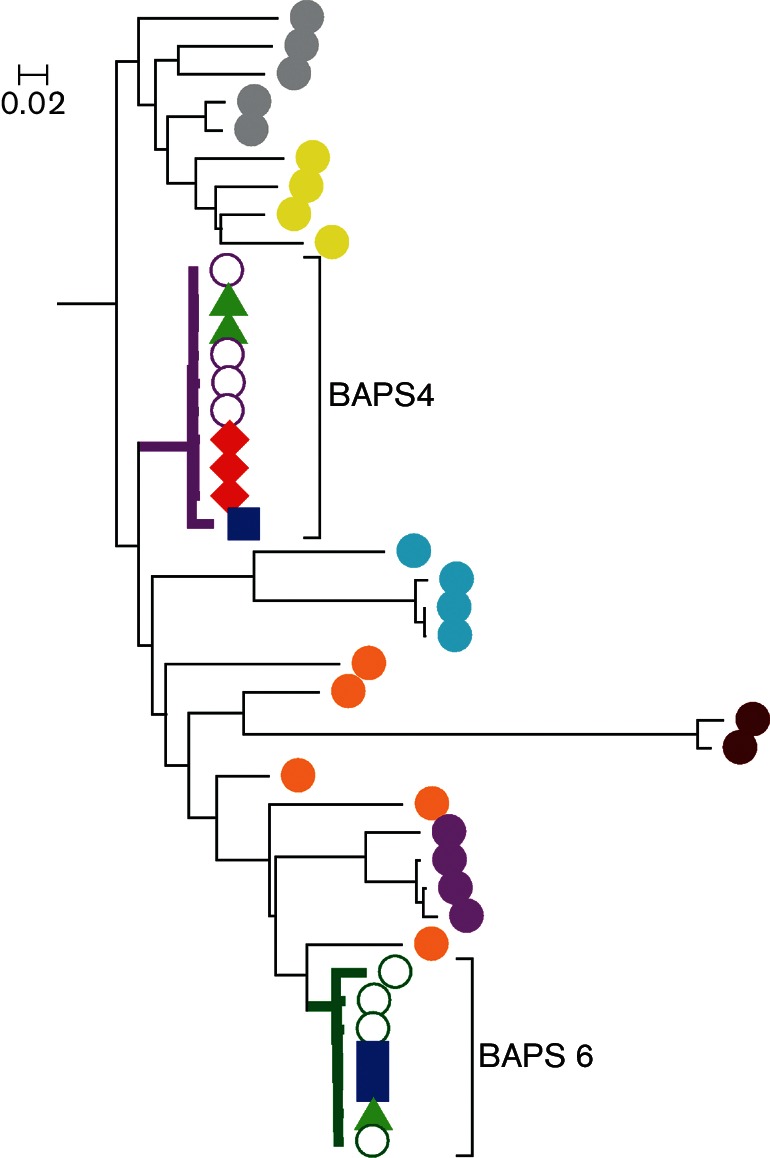
Comparison of ST-45 of Canadian, Italian and barnacle geese origin and one representative of each hierBAPS cluster level 2 on the *b-*lineage. Purple open circle: BAPS 4 isolate, green open circles: BAPS 6 isolate, green triangles: barnacle geese isolate, red diamonds: Canadian strains, blue squares; Italian strains. BAPS 4 and 6 are highlighted. Genetic distance (nt substitutions per site) is indicated with a bar.

To assess a possible global distribution of monomorphic clones and if migrating birds could participate in the dissemination of *C. jejuni* across borders, a rapid core genome phylogenetic tree was reconstructed using one representative of each BAPS level 2 and Dataset two with genomes of *C. jejuni* ST-45 isolates obtained from Italy, Canada and migratory barnacle geese. Two and one geese isolates clustered within the clonal clades of BAPS 4 and 6, respectively (Fig. 4), while three Canadian and one Italian *C. jejuni* from 2011 and 2015, respectively, clustered within the diversity of the clonal BAPS 4 cluster. Moreover, two Italian isolates from 2015 clustered with the BAPS 6 population (Fig. 4). Overall, these data indicate a global dispersion of these monomorphic *C. jejuni* genotypes.

## Discussion

Our study on the genomic diversity of a *C. jejuni* clonal complex across space and time is novel and important in the assessment of molecular epidemiology of campylobacteriosis in the context of public health. By using various state-of-the art methods to search for spatial and temporal signals in isolates of the widespread ST-45 CC, we found an overrepresentation of certain sublineages in the UK and Finland. However, the presence of the geographical signals varied between these sublineages and, when present, followed the clonal frame. In contrast to this, geography and time had no effect on genetic diversity of isolates in two BAPS populations of ST-45. Therefore, we propose that these two ST-45 sublineages are globally circulating monomorphic clones, possibly disseminated by migratory birds as suggested by the additional data on strains from barnacle geese.

The spatial signal in the core genealogy, population structure and accessory genome of ST-45 CC varied between sublineages, while no evidence of a temporal signal was found. The spatial signal, however, was weakly, inconclusively and variably reflected when the data was corrected for population structure, as shown in our GWAS. This supports the hypothesis that the observed genetic separation between Finnish and British strains is most likely to be due to the clonal expansion of specific clades within countries. Genomic separation between the *C. jejuni* populations in Finland and the UK has been noted before (McCarthy *et al.*, 2012), and the authors proposed that the observed separation was due to differences in the epidemiology of campylobacteriosis and to seasonal fluctuations of specific CCs (i.e. ST-45 and ST-283 CCs). In our data, we found that the Finnish monophyletic branch of BAPS 2 corresponds to the expansion of clonal genotype of ST-230, the second most common ST in Finnish patients (Kovanen *et al.*, 2014b) and chickens (Llarena *et al.*, 2015a). In contrast to this, ST-230 is almost non-existent in British human isolates (Cody *et al.*, 2015). This pattern of overrepresentation of certain sublineages according to geography was observed for the British BAPS 7 and 9, and the Baltic BAPS 10, which might reflect either an adaptation of these sublineages to their respective countries or the presence of an ecological barrier.

The major clade *a* consisted entirely of British strains of ST-4791, ST-7259 and ST-7256. These isolates were of non-agricultural animal origin according to the pubMLST database, and a major part of their core genome showed signs of recombination with lineages outside our dataset. This branch uniformly carried the three metabolic accessory genes *ggt,* fucose permease (*fucP)* and a glucose metabolism locus. The combination of *fucP* and *ggt* is rare across the species, while the exclusive presence of *fucP* or *ggt* is common in ST-21 CC and ST-45 CC, respectively (Gripp *et al.*, 2011; de Haan *et al.*, 2012; Zautner *et al.*, 2012). The relatively large genetic distance between clades *a* and *b,* the increased r:m ratio and presence of non-typical genetic features in clade *a* indicate the lack of a shared clonal origin between these clades. This indicates that the clonal definition is unable to correctly summarize the relationship between these isolates. Moreover, a similar incongruence between the genealogy and MLST nomenclature was detected within clade *b* as well, where we frequently observed polyphletic or paraphyletic relationships among members of the same ST. These results complicate the implementation of a hierarchical WGS nomenclature in surveillance and outbreak investigations, as such an approach would deem isolates of different STs unrelated even though their genealogy shows otherwise, as seen, e.g. for the ST-11 isolate (3217-08). According to our analysis, this isolate was more closely related to ST-2219 than other isolates of ST-11, and such a bias could lead to the wrong conclusions in a public health context.

Certain sublineages of BAPS 7 and 10 and the entire BAPS 4, 5, 6 and 11 were of mixed geographical origin, and most of these isolates were ST-45 (96 %). BAPS 4 and BAPS 6 were surprisingly stable over time and space, and both the genetic variation (average 73 SNPs in a 1 Mb non-recombining core genome) and levels of recombination were extremely limited. Moreover, our analysis excluded the presence of a temporal signal within the sampling period of 12 years. Since Canadian, Italian and barnacle geese isolates clustered within the diversity of BAPS 4 and 6, we argue for the existence of at least two globally distributed and temporally stable monomorphic genotypes of ST-45. The spread of genetically monomorphic lineages of bacteria within species of greater diversity have been documented earlier for *E. coli* O157:H7 (Leopold *et al.*, 2009) and *Salmonella enterica* serovar Typhi (Holt *et al.*, 2008). According to Achtman (2012), such population dynamics represent neutral evolution in the form of mild purifying selection in the place of periodic selection of fitter variants, as observed in Darwinian evolution and for *E. coli* under laboratory conditions (Lenski & Travisano, 1994). The author further suggested that the difference in evolution between the experimental and natural populations could be due to bottlenecks imposed on the genetic diversity during zoonotic and geographical transmission (Achtman, 2012). The species *C. jejuni* is a zoonotic pathogen, and ST-45 is a generalist found in a myriad of agricultural animals, wild birds and mammals and has also been isolated from the extra-intestinal environment such as water and sand (Sheppard *et al.*, 2013). Host jumps from animals to humans as well as between animals are most probably frequent and happen at such a pace that the genetic host signature has been eroded in generalist lineages (Dearlove *et al.*, 2015). Therefore, the evolutionary mechanism proposed by Achtman is certainly plausible for ST-45. Future studies geared towards the evolutionary mechanisms and ecological conditions maintaining these clones are needed.

Migrating birds, food trade and people travelling could transmit and disseminate such monomorphic clones globally (French *et al.*, 2014). However, observations of genetic variation over time would still be warranted since studies have estimated a relatively high mutation rate for *C. jejuni* with 23 to 32 SNP generated per 1 Mb per year (Wilson *et al.*, 2009; Achtman, 2012). No temporal signal was evident in any of the sublineages, and *C. jejuni* strains isolated 12 years apart were no more different than those collected during the same year. These results indicate that monomorphic *C. jejuni* populations evolve much more slowly than expected [based on the prediction of Wilson *et al.* (2009)] and suggests that the application of a single mutation rate across the species can be problematic and lead to incorrect prediction of the time of the most recent common ancestor. However, further studies, both longitudinal laboratory studies and observational studies on a wide dataset of varied lineages, geographical origin and longer timespan, could possibly untangle the variability in the evolutionary speed in different *C. jejuni* lineages.

## Conclusion

We have identified problems with the use of a MLST-based hierarchical nomenclature system for *C. jejuni* within ST-45 CC, since the WGS genealogy harbored both polyphyletic and paraphyletic STs, complicating the use of such systems in genomic epidemiology. Furthermore, we show the global occurrence and dissemination of two successful monomorphic clones of ST-45 and describe a national clonal expansion of Finnish ST-230, and predict that other monomorphic clones of *C. jejuni* will be discovered as the number of WGS studies increases. The evolutionary mechanisms and ecological conditions maintaining these clones are not known, and further research into this area is needed. The occurrence of monomorphic clones represents a problem for genomic epidemiology in surveillance and monitoring, as even WGS lacks sufficient capacity to reliably differentiate between these extremely similar isolates. Our results on the occurrence of monomorphic clones among *C. jejuni* highlight the importance of two principles in genomic epidemiology; we can only exclude the possibility that isolates are epidemiologically linked with a considerable level of certainty and that the combination of genomic and epidemiological data will be crucial for use of WGS as a reliable and stable working tool in public health.

## Supplementary Data

584 Supplementary File 1

## References

[R4] Abascal F. Zardoya R. Telford M. J.(2010). TranslatorX: multiple alignment of nucleotide sequences guided by amino acid translations. Nucleic Acids Res 38 W7–13. 10.1093/nar/gkq291 20435676 PMC2896173

[R5] Achtman M.(2012). Insights from genomic comparisons of genetically monomorphic bacterial pathogens. Philos Trans R Soc Lond B Biol Sci 367 860–867. 10.1098/rstb.2011.0303 22312053 PMC3267118

[R6] Altschul S. F. Madden T. L. Schäffer A. A. Zhang J. Zhang Z. Miller W. Lipman D. J.(1997). Gapped blast and PSI-blast: a new generation of protein database search programs. Nucleic Acids Res 25 3389–3402. 10.1093/nar/25.17.3389 9254694 PMC146917

[R7] Aziz R. K. Bartels D. Best A. A. DeJongh M. Disz T. Edwards R. A. Formsma K. Gerdes S. Glass E. M.(2008). The RAST Server: rapid annotations using subsystems technology. BMC Genomics 9 9–75. 10.1186/1471-2164-9-75 18261238 PMC2265698

[R9] Bankevich A. Nurk S. Antipov D. Gurevich A. A. Dvorkin M. Kulikov A. S. Lesin V. M. Nikolenko S. I. Pham S.(2012). SPAdes: a new genome assembly algorithm and its applications to single-cell sequencing. J Comput Biol 19 455–47. 10.1089/cmb.2012.0021 22506599 PMC3342519

[R10] Batchelor R. A. Pearson B. M. Friis L. M. Guerry P. Wells J. M.(2004). Nucleotide sequences and comparison of two large conjugative plasmids from different *Campylobacter* species. Microbiology 150 3507–3517. 10.1099/mic.0.27112-0 15470128

[R11] Blaser M. Engberg J.(2008). Clinical aspects of *Campylobacter jejuni* and *Campylobacter coli* infections. Campylobacter 99–121. Edited by Nachamkin I. Szymanski C. Blaser M. J. Washington, DC: ASM Press.

[R12] Cheng L. Connor T. R. Sirén J. Aanensen D. M. Corander J.(2013). Hierarchical and spatially explicit clustering of DNA sequences with BAPS software. Mol Biol Evol 30 1224–1228. 10.1093/molbev/mst028 23408797 PMC3670731

[R13] Ciccarelli F. D. Doerks T. von Mering C. Creevey C. J. Snel B. Bork P.(2006). Toward automatic reconstruction of a highly resolved tree of life. Science 311 1283–1287. 10.1126/science.1123061 16513982

[R14] Cody A. J. McCarthy N. D. Bray J. E. Wimalarathna H. M. Colles F. M. Jansen van Rensburg M. J. Dingle K. E. Waldenström J. Maiden M. C.(2015). Wild bird-associated *Campylobacter jejuni* isolates are a consistent source of human disease, in Oxfordshire, United Kingdom. Environ Microbiol Rep 7 782–788. 10.1111/1758-2229.12314 26109474 PMC4755149

[R15] de Haan C. P. Kivistö R. Hakkinen M. Rautelin H. Hänninen M. L.(2010). Decreasing trend of overlapping multilocus sequence types between human and chicken *Campylobacter jejuni* isolates over a decade in Finland. Appl Environ Microbiol 76 5228–5236. 10.1128/AEM.00581-10 20543048 PMC2916457

[R16] de Haan C. P. Llarena A. K. Revez J. Hänninen M. L.(2012). Association of *Campylobacter jejuni* metabolic traits with multilocus sequence types. Appl Environ Microbiol 78 5550–5554. 10.1128/AEM.01023-12 22660710 PMC3406166

[R17] Dearlove B. Cody A. Pascoe B. Meric G. Wilson D. Sheppard S.(2015). Rapid host switching in generalist *Campylobacter* strains erodes the signal for tracing human infections. ISME Journal 10 721–729. 26305157 10.1038/ismej.2015.149 PMC4677457

[R18] Dingle K. E. Colles F. M. Wareing D. R. Ure R. Fox A. J. Bolton F. E. Bootsma H. J. Willems R. J. Urwin R. Maiden M. C.(2001). Multilocus sequence typing system for *Campylobacter jejuni*. J Clin Microbiol 39 14–23. 10.1128/JCM.39.1.14-23.2001 11136741 PMC87672

[R19] Dingle K. E. Colles F. M. Ure R. Wagenaar J. A. Duim B. Bolton F. J. Fox A. J. Wareing D. R. Maiden M. C.(2002). Molecular characterization of *Campylobacter jejuni* clones: a basis for epidemiologic investigation. Emerg Infect Dis 8 949–955. 10.3201/eid0809.02-0122 12194772 PMC2732546

[R20] Drummond A. J. Suchard M. A. Xie D. Rambaut A.(2012). Bayesian phylogenetics with BEAUti and the BEAST 1.7. Mol Biol Evol 29 1969–1973. 10.1093/molbev/mss075 22367748 PMC3408070

[R21] Drummond A. J. Bouckaert R. R.(2015). Bayesian Evolutionary Analysis with BEAST, 1st edn Cambridge, UK: University Printing House.

[R22] Edgar R. C.(2004a). muscle: a multiple sequence alignment method with reduced time and space complexity. BMC Bioinformatics 5 113. 15318951 10.1186/1471-2105-5-113 PMC517706

[R23] Edgar R. C.(2004b). muscle: multiple sequence alignment with high accuracy and high throughput. Nucleic Acids Res 32 1792–1797. 10.1093/nar/gkh340 15034147 PMC390337

[R24] Ekseth O. K. Kuiper M. Mironov V.(2014). orthAgogue: an agile tool for the rapid prediction of orthology relations. Bioinformatics 30 734–736. 10.1093/bioinformatics/btt582 24115168

[R25] Enright A. J. Van Dongen S. Ouzounis C. A.(2002). An efficient algorithm for large-scale detection of protein families. Nucleic Acids Res 30 1575–1584. 10.1093/nar/30.7.1575 11917018 PMC101833

[R2] European Centre for Disease Prevention and Control (ECDC)(2015). Expert Opinion on the introduction of next-generation typing methods for food- and waterborne diseases in the EU and EEA. Stockholm, Sweden: ECDC.

[R3] European Food Safety Authority (EFSA) & European Centre for Disease Prevention and Control (ECDC)(2015). The European Union summary report on antimicrobial resistance in zoonotic and indicator bacteria from humans, animals and food in 2013. EFSA J 13 4036. 10.2903/j.efsa.2017.4694 PMC7009883 32625402

[R26] European Food Safety Authority (EFSA)(2010). Analysis of the baseline survey on the prevalence of *Campylobacter* in broiler batches and of *Campylobacter* and *Salmonella* on broiler carcasses in the EU, 2008 – part A: *Campylobacter* and *Salmonella* prevalence estimates. EFSA Journal 8 1503.

[R1] European Food Safety Authority (EFSA)(2014). EFSA Scientific colloquium summary report on use of whole-genome sequencing (WGS) of food-borne pathogens for public health protection. Parma, Italy: EFSA.

[R27] Fouts D. E. Mongodin E. F. Mandrell R. E. Miller W. G. Rasko D. A. Ravel J. Brinkac L. M. DeBoy R. T. Parker C. T.(2005). Major structural differences and novel potential virulence mechanisms from the genomes of multiple *Campylobacter* species. PLoS Biol 3,e15. 10.1371/journal.pbio.0030015 15660156 PMC539331

[R28] French N. Yu S. Biggs P. Holland B. Fearnhead P. Binney B. Fox A. Grove-White D. Leigh J.(2014). Evolution of *Campylobacter* species in New Zealand. Campylobacter Ecology and Evolution 221–204. Edited by Sheppard S. Meric G. Swansea, UK: Caister Academic Press.

[R29] Friis C. Wassenaar T. M. Javed M. A. Snipen L. Lagesen K. Hallin P. F. Newell D. G. Toszeghy M. Ridley A.(2010). Genomic characterization of *Campylobacter jejuni* strain M1. *PloS one*.5,e12253. 10.1371/journal.pone.0012253 PMC2928727 20865039

[R30] Gripp E. Hlahla D. Didelot X. Kops F. Maurischat S. Tedin K. Alter T. Ellerbroek L. Schreiber K.(2011). Closely related *Campylobacter jejuni* strains from different sources reveal a generalist rather than a specialist lifestyle. BMC Genomics 12 584. 10.1186/1471-2164-12-584 22122991 PMC3283744

[R31] Habib I. Louwen R. Uyttendaele M. Houf K. Vandenberg O. Nieuwenhuis E. E. Miller W. G. van Belkum A. De Zutter L.(2009). Correlation between genotypic diversity, lipooligosaccharide gene locus class variation, and caco-2 cell invasion potential of *Campylobacter jejuni* isolates from chicken meat and humans: contribution to virulotyping. Appl Environ Microbiol 75 4277–4288. 10.1128/AEM.02269-08 19411422 PMC2704853

[R32] Hammer Ø. Harper D. A. T. Ryan P. D.(2001). PAST: paleontological statistics software package for education and data analysis. Palaeontol. Elect 4 1–9.

[R33] Hasegawa M. Kishino H. Yano T.(1985). Dating of the human–ape splitting by a molecular clock of mitochondrial DNA. J Mol Evol 22 160–174. 10.1007/BF02101694 3934395

[R34] Hendriksen R. S. Price L. B. Schupp J. M. Gillece J. D. Kaas R. S. Engelthaler D. M. Bortolaia V. Pearson T. Waters A. E.(2011). Population genetics of *Vibrio cholerae* from Nepal in 2010: evidence on the origin of the Haitian outbreak. MBio 2,e00157–11. 10.1128/mBio.00157-11 21862630 PMC3163938

[R35] Hofreuter D. Tsai J. Watson R. O. Novik V. Altman B. Benitez M. Clark C. Perbost C. Jarvie T.(2006). Unique features of a highly pathogenic *Campylobacter jejuni* strain. Infect Immun 74 4694–4707. 10.1128/IAI.00210-06 16861657 PMC1539605

[R36] Hofreuter D. Novik V. Galán J. E.(2008). Metabolic diversity in *Campylobacter jejuni* enhances specific tissue colonization. Cell Host Microbe 4 425–433. 10.1016/j.chom.2008.10.002 18996343

[R37] Holt K. E. Parkhill J. Mazzoni C. J. Roumagnac P. Weill F. X. Goodhead I. Rance R. Baker S. Maskell D. J.(2008). High-throughput sequencing provides insights into genome variation and evolution in *Salmonella* Typhi. Nat Genet 40 987–993. 10.1038/ng.195 18660809 PMC2652037

[R38] Jaakola S. Lyytikäinen O. Huusko S. Salmenlinna S. Pirohonen J. Savolainen- Korpa C. Liitsola K. Jalava J. Toropainen M.(2015). Tartuntataudit Suomessa 2014. THL Reports 11.

[R39] Jolley K. A. Maiden M. C.(2010). BIGSdb: scalable analysis of bacterial genome variation at the population level. BMC Bioinformatics 11 595. 10.1186/1471-2105-11-595 21143983 PMC3004885

[R40] Kivistö R. I. Kovanen S. Skarp-de Haan A. Schott T. Rahkio M. Rossi M. Hänninen M. L.(2014). Evolution and comparative genomics of *Campylobacter jejuni* ST-677 clonal complex. Genome Bbiol Evol 6 2424–2438. 10.1093/gbe/evu194 PMC4202330 25193305

[R44] Kovanen S. Kivistö R. Llarena A. K. Zhang J. Kärkkäinen U. M. Tuuminen T. Uksila J. Hakkinen M. Rossi M.(2016). Tracing isolates from domestic human *Campylobacter jejuni* infections to chicken slaughter batches and swimming water using whole-genome multilocus sequence typing. Int J Food Microbiol 226 53–60. 10.1016/j.ijfoodmicro.2016.03.009 27041390

[R42] Kovanen S. M. Kivistö R. I. Rossi M. Hänninen M.-L.(2014a). A combination of MLST and CRISPR typing reveals dominant *Campylobacter jejuni* types in organically farmed laying hens. J Appl Microbiol 117 249–257. 10.1111/jam.12503 24655229

[R43] Kovanen S. M. Kivisto R. I. Rossi M. Schott T. Karkkainen U. M. Tuuminen T. Uksila J. Rautelin H. Hänninen M.-L.(2014b). Multilocus sequence typing (MLST) and whole-genome MLST of *Campylobacter jejuni* isolates from human infections in three districts during a seasonal peak in Finland. J Clin Microbiol 52 4147–4154. 10.1128/JCM.01959-14 25232158 PMC4313278

[R45] Kuo C. H. Ochman H.(2009). Inferring clocks when lacking rocks: the variable rates of molecular evolution in bacteria. Biol Direct 4 35. 10.1186/1745-6150-4-35 19788732 PMC2760517

[R46] Kärenlampi R. Rautelin H. Schönberg-Norio D. Paulin L. Hänninen M. L.(2007). Longitudinal study of finnish *Campylobacter jejuni* and *C. coli* isolates from humans, using multilocus sequence typing, including comparison with epidemiological data and isolates from poultry and cattle. Appl Environ Microbiol 73 148–155. 10.1128/AEM.01488-06 17085689 PMC1797135

[R41] Köser C. U. Holden M. T. Ellington M. J. Cartwright E. J. Brown N. M. Ogilvy-Stuart A. L. Hsu L. Y. Chewapreecha C. Croucher N. J.(2012). Rapid whole-genome sequencing for investigation of a neonatal MRSA outbreak. N Engl J Med 366 2267–2275. 10.1056/NEJMoa1109910 22693998 PMC3715836

[R47] Lenski R. E. Travisano M.(1994). Dynamics of adaptation and diversification: a 10 000-generation experiment with bacterial populations. Proc Natl Acad Sci U S A 91 6808–6814. 10.1073/pnas.91.15.6808 8041701 PMC44287

[R48] Leopold S. R. Magrini V. Holt N. J. Shaikh N. Mardis E. R. Cagno J. Ogura Y. Iguchi A. Hayashi T.(2009). A precise reconstruction of the emergence and constrained radiations of *Escherichia coli* O157 portrayed by backbone concatenomic analysis. Proc Natl Acad Sci U S A 106 8713–8718. 10.1073/pnas.0812949106 19439656 PMC2689004

[R49] Linz B. Balloux F. Moodley Y. Manica A. Liu H. Roumagnac P. Falush D. Stamer C. Prugnolle F.(2007). An African origin for the intimate association between humans and *Helicobacter pylori*. Nature 445 915–918. 10.1038/nature05562 17287725 PMC1847463

[R51] Llarena A. K. Skarp-de Haan C. P. Rossi M. Hänninen M. L.(2015). Characterization of the *Campylobacter jejuni* population in the barnacle geese reservoir. Zoonoses Public Health 62 209–221. 10.1111/zph.12141 24948379

[R50] Llarena A. K. Huneau A. Hakkinen M. Hänninen M. L.(2015a). Predominant *Campylobacter jejuni* sequence types persist in finnish chicken production. PLoS One 10,e0116585 10.1371/journal.pone.0116585 25700264 PMC4336332

[R52] Marttinen P. Hanage W. P. Croucher N. J. Connor T. R. Harris S. R. Bentley S. D. Corander J.(2012). Detection of recombination events in bacterial genomes from large population samples. Nucleic Acids Res 40,e6. 10.1093/nar/gkr928 22064866 PMC3245952

[R53] McCarthy N. D. Gillespie I. A. Lawson A. J. Richardson J. Neal K. R. Hawtin P. R. Maiden M. C. O'Brien S. J.(2012). Molecular epidemiology of human *Campylobacter jejuni* shows association between seasonal and international patterns of disease. Epidemiol Infect 140 2247–2255. 10.1017/S0950268812000192 22370165 PMC3487483

[R54] Morelli G. Didelot X. Kusecek B. Schwarz S. Bahlawane C. Falush D. Suerbaum S. Achtman M.(2010). Microevolution of *Helicobacter pylori* during prolonged infection of single hosts and within families. PLoS Genet 6,e1001036. 10.1371/journal.pgen.1001036 20661309 PMC2908706

[R55] Müllner P. Collins-Emerson J. M. Midwinter A. C. Carter P. Spencer S. E. van der Logt P. Hathaway S. French N. P.(2010). Molecular epidemiology of *Campylobacter jejuni* in a geographically isolated country with a uniquely structured poultry industry. Appl Environ Microbiol 76 2145–2154. 10.1128/AEM.00862-09 20154115 PMC2849254

[R74] Rambaut A. Lam T. T. Max Carvalho L. Pybus O. G.(2016). Exploring the temporal structure of heterochronous sequences using TempEst (formerly Path-O-Gen). Virus Evolution 2 vew007 10.1093/ve/vew007 27774300 PMC4989882

[R56] Reeves P. R. Liu B. Zhou Z. Li D. Guo D. Ren Y. Clabots C. Lan R. Johnson J. R.(2011). Rates of mutation and host transmission for an *Escherichia coli* clone over 3 years. PLoS One 6,e26907. 10.1371/journal.pone.0026907 22046404 PMC3203180

[R72] Revez J. Hänninen M. L.(2012). Lipooligosaccharide locus classes are associated with certain Campylobacter jejuni multilocus sequence types. Eur J Clin Microbiol Infect Dis 31 2203–2209. 10.1007/s10096-012-1556-3 22298242

[R57] Revez J. Llarena A. K. Schott T. Kuusi M. Hakkinen M. Kivistö R. Hänninen M. L. Rossi M.(2014a). Genome analysis of *Campylobacter jejuni* strains isolated from a waterborne outbreak. BMC Genomics 15 768 10.1186/1471-2164-15-768 25196593 PMC4168118

[R58] Revez J. Zhang J. Schott T. Kivistö R. Rossi M. Hänninen M. L.(2014b). Genomic variation between milkborne outbreak-associated *Campylobacter jejuni* isolates. J Clin Microbiol 52 2782–2786. 24850348 10.1128/JCM.00931-14 PMC4136164

[R60] Sheppard S. K. Didelot X. Meric G. Torralbo A. Jolley K. A. Kelly D. J. Bentley S. D. Maiden M. C. Parkhill J.(2013). Genome-wide association study identifies vitamin B5 biosynthesis as a host specificity factor in *Campylobacter*. Proc Natl Acad Sci U S A 110 11923–11927. 10.1073/pnas.1305559110 23818615 PMC3718156

[R61] Sheppard S. K. Cheng L. Méric G. de Haan C. P. Llarena A. K. Marttinen P. Vidal A. Ridley A. Clifton-Hadley F.(2014). Cryptic ecology among host generalist *Campylobacter jejuni* in domestic animals. Mol Ecol 23 2442–2451. 10.1111/mec.12742 24689900 PMC4237157

[R62] Skarp C. P. Akinrinade O. Nilsson A. J. Ellström P. Myllykangas S. Rautelin H.(2015). Comparative genomics and genome biology of invasive *Campylobacter jejuni*. Sci Rep 5 17300. 10.1038/srep17300 26603914 PMC4658567

[R63] Smith E. E. Buckley D. G. Wu Z. Saenphimmachak C. Hoffman L. R. D'Argenio D. A. Miller S. I. Ramsey B. W. Speert D. P.(2006). Genetic adaptation by *Pseudomonas aeruginosa* to the airways of cystic fibrosis patients. Proc Natl Acad Sci U S A 103 8487–8492. 10.1073/pnas.0602138103 16687478 PMC1482519

[R64] Stamatakis A.(2014). RAxML version 8: a tool for phylogenetic analysis and post-analysis of large phylogenies. Bioinformatics 30 1312–1313. 10.1093/bioinformatics/btu033 24451623 PMC3998144

[R65] Taboada E. N. Mackinnon J. M. Luebbert C. C. Gannon V. P. Nash J. H. Rahn K.(2008). Comparative genomic assessment of multi-locus sequence typing: rapid accumulation of genomic heterogeneity among clonal isolates of *Campylobacter jejuni*. BMC Evol Biol 8 229. 10.1186/1471-2148-8-229 18691421 PMC2527321

[R66] Tamura K. Peterson D. Peterson N. Stecher G. Nei M. Kumar S.(2011). mega5: molecular evolutionary genetics analysis using maximum likelihood, evolutionary distance, and maximum parsimony methods. Mol Biol Evol 28 2731–2739. 10.1093/molbev/msr121 21546353 PMC3203626

[R67] Treangen T. J. Ondov B. D. Koren S. Phillippy A. M.(2014). The Harvest suite for rapid core-genome alignment and visualization of thousands of intraspecific microbial genomes. Genome Biol 15 524–539. 10.1186/s13059-014-0524-x 25410596 PMC4262987

[R68] Vorwerk H. Huber C. Mohr J. Bunk B. Bhuju S. Wensel O. Spröer C. Fruth A. Flieger A.(2015). A transferable plasticity region in *Campylobacter coli* allows isolates of an otherwise non-glycolytic food-borne pathogen to catabolize glucose. Mol Microbiol 98 809–830. 10.1111/mmi.13159 26259566

[R73] Weinert L. A. Chaudhuri R. R. Wang J. Peters S. E. Corander J. Jombart T. Baig A. Howell K. J. Vehkala M.(2015). Erratum: genomic signatures of human and animal disease in the zoonotic pathogen Streptococcus suis. Nat Commun 6. 10.1038/ncomms8272 25966020 PMC4462838

[R69] Wilson D. J. Gabriel E. Leatherbarrow A. J. Cheesbrough J. Gee S. Bolton E. Fox A. Hart C. A. Diggle P. J.(2009). Rapid evolution and the importance of recombination to the gastroenteric pathogen *Campylobacter jejuni*. Mol Biol Evol 26 385–397. 10.1093/molbev/msn264 19008526 PMC2639114

[R70] Zautner A. E. Ohk C. Tareen A. M. Lugert R. Gross U.(2012). Epidemiological association of *Campylobacter jejuni* groups with pathogenicity-associated genetic markers. BMC Microbiol 12 171–180. 10.1186/1471-2180-12-171 22873291 PMC3487957

[R71] Zhang J. Halkilahti J. Hänninen M. L. Rossi M.(2015). Refinement of whole-genome multilocus sequence typing analysis by addressing gene paralogy. J Clin Microbiol 53 1765–1767. 10.1128/JCM.00051-15 25788543 PMC4400783

