# Supplementary material for: Monomorphic genotypes within a generalist lineage of Campylobacter jejuni show signs of global dispersion
Source: Microb Genom. 2016 Oct 21;2(10):e000088. doi: 10.1099/mgen.0.000088 (PMC5359405; doi:10.1099/mgen.0.000088)
Supplement: Supplementary file 1 [file mgen-02-88-s001.pdf]

Isolate	ST	CC	Country	Year	BAPS L1	BAPS L2	Source	ID number PubMLST	ENA accession number	Study
Dataset one										
**Dg20a**	7259	45	UK	UNK	1	1	animal	25937		Pearson et al. (2015)
**Dg26b**	7259	45	UK	UNK	1	1	animal	25959		Pearson et al. (2015)
**Dg95**	7259	45	UK	UNK	1	1	animal	26020		Pearson et al. (2015)
**Dg137**	7259	45	UK	UNK	1	1	animal	26031		Pearson et al. (2015)
**Dg246**	7259	45	UK	UNK	1	1	animal	26063		Pearson et al. (2015)
**Dg63a**	7259	45	UK	UNK	1	2	animal	25975		Pearson et al. (2015)
**Dg122**	4791	45	UK	UNK	1	3	animal	25999		Pearson et al. (2015)
**Dg180**	4791	45	UK	UNK	1	3	animal	26034		Pearson et al. (2015)
**Dg184**	4791	45	UK	UNK	1	3	animal	26040		Pearson et al. (2015)
**Dg187**	4791	45	UK	UNK	1	3	animal	26054		Pearson et al. (2015)
**8623-12**	583	45	Finland	2012	2	5	animal	47996		Llarena et al. (2015)
**8624-12**	583	45	Finland	2012	2	5	animal	48011		Llarena et al. (2015)
**OXC4559**	583	45	UK	2009	2	5	human	24039		Oxfordshire Human Surveillance Study
**OXC4850**	583	45	UK	2010	2	5	human	22163		Oxfordshire Human Surveillance Study
**OXC6294**	583	45	UK	2011	2	5	human	16091		Oxfordshire Human Surveillance Study
**OXC6330**	583	45	UK	2011	2	5	human	16127		Oxfordshire Human Surveillance Study
**OXC6819**	583	45	UK	2012	2	5	human	18376		Oxfordshire Human Surveillance Study
**OXC7584**	583	45	UK	2012	2	5	human	24518		Oxfordshire Human Surveillance Study
**OXC7587**	583	45	UK	2013	2	5	human	24521		Oxfordshire Human Surveillance Study
**OXC8080**	583	45	UK	2013	2	5	human	25519		Oxfordshire Human Surveillance Study
**Dg24**	583	45	UK	UNK	2	5	animal	25944		Pearson et al. (2015)
**Dg25a**	583	45	UK	UNK	2	5	animal	25955		Pearson et al. (2015)
**Dg28a**	583	45	UK	UNK	2	5	animal	25963		Pearson et al. (2015)

**Dg366**	583	45	UK	UNK	2	5	animal	26013		Pearson et al. (2015)
**Dg260**	583	45	UK	UNK	2	5	animal	26051		Pearson et al. (2015)
**OXC5091**	583	45	UK	2010	2	6	human	24732		Oxfordshire Human Surveillance Study
**OXC5224**	583	45	UK	2010	2	6	human	24810		Oxfordshire Human Surveillance Study
**OXC6970**	583	45	UK	2012	2	6	human	21144		Oxfordshire Human Surveillance Study
**OXC7032**	583	45	UK	2012	2	6	human	22218		Oxfordshire Human Surveillance Study
**OXC8128**	583	45	UK	2013	2	6	human	25459		Oxfordshire Human Surveillance Study
**OXC7344**	334	45	UK	2012	2	7	human	23884		Oxfordshire Human Surveillance Study
**OXC7346**	334	45	UK	2012	2	7	human	23886		Oxfordshire Human Surveillance Study
**OXC8062**	334	45	UK	2013	2	7	human	25512		Oxfordshire Human Surveillance Study
**OXC8055**	334	45	UK	2013	2	7	human	25600		Oxfordshire Human Surveillance Study
**2661-04**	230	45	Finland	2004	2	4	animal	48000		Llarena et al. (2015)
**5441-04**	230	45	Finland	2004	2	4	animal	48024		Llarena et al. (2015)
**5442-04**	230	45	Finland	2004	2	4	animal	48023		Llarena et al. (2015)
**2912-08**	230	45	Finland	2008	2	4	animal	48005		Llarena et al. (2015)
**2913-08**	230	45	Finland	2008	2	4	animal	48019		Llarena et al. (2015)
**J15**	230	45	Finland	2012	2	4	human	46045		Kovanen et al. (2014b)
**J18**	230	45	Finland	2012	2	4	human	46046		Kovanen et al. (2014b)
**J14**	230	45	Finland	2012	2	4	human	46043		Kovanen et al. (2014b)
**K8**	230	45	Finland	2012	2	4	human	46048		Kovanen et al. (2014b)
**K9**	230	45	Finland	2012	2	4	human	46049		Kovanen et al. (2014b)
**S23-1**	230	45	Finland	2012	2	4	environment	46056		Kovanen et al. (2016)
**M4**	230	45	Finland	2012	2	4	human	46055		Kovanen et al. (2014b)
**6175-12**	230	45	Finland	2012	2	4	animal	46040		Llarena et al. (2015)
**6737-12**	230	45	Finland	2012	2	4	animal	46042		Llarena et al. (2015)
**J16**	230	45	Finland	2012	2	4	human	46044		Kovanen et al. (2014b)

**J17**	230	45	Finland	2012	2	4	human	46047		Kovanen et al. (2014b)
**K13**	230	45	Finland	2012	2	4	human	46053		Kovanen et al. (2014b)
**K10**	230	45	Finland	2012	2	4	human	46050		Kovanen et al. (2014b)
**K11**	230	45	Finland	2012	2	4	human	46051		Kovanen et al. (2014b)
**K12**	230	45	Finland	2012	2	4	human	46052		Kovanen et al. (2014b)
**K14**	230	45	Finland	2012	2	4	human	46054		Kovanen et al. (2014b)
**OXC8046**	230	45	UK	2013	2	4	human	25508		Oxfordshire Human Surveillance Study
**FI15**	21	21	Estonia	2012	3	8	animal	46132	PRJEB15115	This study
**7-1C**	45	45	Finland	1999	4	9	animal	46108	PRJEB15115	This study
**BRTI**	45	45	Finland	1999	4	9	animal	46109	PRJEB15115	This study
**UoH4028**	45	45	Finland	2003	4	9	animal	2681		Zhang et al. (2015)
**3721-04**	45	45	Finland	2004	4	9	animal	48017		Llarena et al. (2015)
**2445-08**	45	45	Finland	2008	4	9	animal	48012		Llarena et al. (2015)
**3222-08**	45	45	Finland	2008	4	9	animal	48006		Llarena et al. (2015)
**11962-12**	45	45	Finland	2012	4	9	animal	46014		Llarena et al. (2015)
**5894-12**	45	45	Finland	2012	4	9	animal	45986		Llarena et al. (2015)
**5913-12**	45	45	Finland	2012	4	9	animal	45987		Llarena et al. (2015)
**6390-12**	45	45	Finland	2012	4	9	animal	45995		Llarena et al. (2015)
**6391-12**	45	45	Finland	2012	4	9	animal	45996		Llarena et al. (2015)
**6392-12**	45	45	Finland	2012	4	9	animal	45997		Llarena et al. (2015)
**6393-12**	45	45	Finland	2012	4	9	animal	45998		Llarena et al. (2015)
**6395-12**	45	45	Finland	2012	4	9	animal	45999		Llarena et al. (2015)
**6544-12**	45	45	Finland	2012	4	9	animal	46006		Llarena et al. (2015)
**6545-12**	45	45	Finland	2012	4	9	animal	46007		Llarena et al. (2015)
**6546-12**	45	45	Finland	2012	4	9	animal	46008		Llarena et al. (2015)
**J6**	45	45	Finland	2012	4	9	human	46019		Kovanen et al. (2014b)
**J9**	45	45	Finland	2012	4	9	human	46022		Kovanen et al. (2014b)
**J12**	45	45	Finland	2012	4	9	human	46025		Kovanen et al. (2014b)
**J4**	45	45	Finland	2012	4	9	human	46017		Kovanen et al. (2014b)
**J10**	45	45	Finland	2012	4	9	human	46023		Kovanen et al. (2014b)

**J7**	45	45	Finland	2012	4	9	human	46020		Kovanen et al. (2014b)
**J5**	45	45	Finland	2012	4	9	human	46018		Kovanen et al. (2014b)
**J2**	45	45	Finland	2012	4	9	human	46015		Kovanen et al. (2014b)
**J13**	45	45	Finland	2012	4	9	human	46026		Kovanen et al. (2014b)
**J3**	45	45	Finland	2012	4	9	human	46016		Kovanen et al. (2014b)
**K1**	45	45	Finland	2012	4	9	human	46027		Kovanen et al. (2014b)
**OXC4893**	45	45	UK	2010	4	9	human	24459		Oxfordshire Human Surveillance Study
**OXC6321**	45	45	UK	2011	4	9	human	16118		Oxfordshire Human Surveillance Study
**OXC6536**	45	45	UK	2011	4	9	human	16328		Oxfordshire Human Surveillance Study
**OXC6797**	45	45	UK	2012	4	9	human	18354		Oxfordshire Human Surveillance Study
**OXC7129**	45	45	UK	2012	4	9	human	21210		Oxfordshire Human Surveillance Study
**OXC7284**	45	45	UK	2012	4	9	human	22325		Oxfordshire Human Surveillance Study
**OXC7745**	45	45	UK	2013	4	9	human	24980		Oxfordshire Human Surveillance Study
**OXC8238**	45	45	UK	2013	4	9		27939		Oxfordshire Human Surveillance Study
**OXC7216**	45	45	UK	2012	4	9	human	22707		Oxfordshire Human Surveillance Study
**OXC7233**	45	45	UK	2012	4	9	human	22723		Oxfordshire Human Surveillance Study
**K6**	45	45	Finland	2012	4	10	human	46032		Kovanen et al. (2014b)
**S28-1**	45	45	Finland	2012	4	10	environment	46038		Kovanen et al. (2016)
**FI25**	45	45	Estonia	2012	4	11	animal	46137	PRJEB15115	This study
**K4**	45	45	Finland	2012	4	12	human	46030		Kovanen et al. (2014b)
**FI39**	45	45	Lithuania	2012	5	13	animal	46135	PRJEB15115	This study
**3485-08**	45	45	Finland	2008	5	15	animal	47999		Llarena et al. (2015)
**M3**	45	45	Finland	2012	5	15	human	46035		Kovanen et al. (2014b)
**5760-12**	45	45	Finland	2012	5	15	animal	45983		Llarena et al. (2015)
**5761-12**	45	45	Finland	2012	5	15	animal	45984		Llarena et al. (2015)

**5762-12**	45	45	Finland	2012	5	15	animal	45985		Llarena et al. (2015)
**6179-12**	45	45	Finland	2012	5	15	animal	45989		Llarena et al. (2015)
**J11**	45	45	Finland	2012	5	15	human	46024		Kovanen et al. (2014b)
**S17-1**	45	45	Finland	2012	5	15	environment	46037		Kovanen et al. (2016)
**OXC5420**	45	45	UK	2010	5	15	human	21372		Oxfordshire Human Surveillance Study
**OXC6437**	45	45	UK	2011	5	15	human	16232		Oxfordshire Human Surveillance Study
**OXC7061**	45	45	UK	2012	5	15	human	22245		Oxfordshire Human Surveillance Study
**OXC7267**	45	45	UK	2012	5	15	human	22308		Oxfordshire Human Surveillance Study
**OXC7186**	45	45	UK	2012	5	15	human	22677		Oxfordshire Human Surveillance Study
**OXC7484**	45	45	UK	2012	5	15	human	24012		Oxfordshire Human Surveillance Study
**OXC7793**	45	45	UK	2013	5	15	human	24596		Oxfordshire Human Surveillance Study
**OXC7735**	45	45	UK	2013	5	15	human	24973		Oxfordshire Human Surveillance Study
**OXC8065**	45	45	UK	2013	5	15	human	25515		Oxfordshire Human Surveillance Study
**OXC8228**	45	45	UK	2013	5	15		27930		Oxfordshire Human Surveillance Study
**Dg145**	45	45	UK	UNK	5	15	animal	24193		Pearson et al. (2015)
**Dg162**	45	45	UK	UNK	5	15	animal	26024		Pearson et al. (2015)
**Dg282_R**	45	45	UK	UNK	5	15	animal	28057		Pearson et al. (2015)
**S16-1**	45	45	Finland	2012	5	16	environment	46036		Kovanen et al. (2016)
**OXC5180**	45	45	UK	2010	5	14	human	24777		Oxfordshire Human Surveillance Study
**OXC8072**	45	45	UK	2013	5	14	human	25606		Oxfordshire Human Surveillance Study
**OXC7708**	45	45	UK	2013	5	14	human	25629		Oxfordshire Human Surveillance Study
**UoHIHV116260**	45	45	Finland	2000	6	17	human	2693		Revez et al. (2014)
**3455-04**	45	45	Finland	2004	6	17	animal	48021		Llarena et al. (2015)
**3461-04**	45	45	Finland	2004	6	17	animal	48013		Llarena et al. (2015)

**3578-04**	45	45	Finland	2004	6	17	animal	48030		Llarena et al. (2015)
**49-8S**	45	45	Finland	2004	6	17	animal	46110	PRJEB15115	This study
**49-9S**	45	45	Finland	2004	6	17	animal	46111	PRJEB15115	This study
**3976-08**	45	45	Finland	2008	6	17	animal	48010		Llarena et al. (2015)
**3977-08**	45	45	Finland	2008	6	17	animal	48002		Llarena et al. (2015)
**3978-08**	45	45	Finland	2008	6	17	animal	48016		Llarena et al. (2015)
**3979-08**	45	45	Finland	2008	6	17	animal	48027		Llarena et al. (2015)
**3980-08**	45	45	Finland	2008	6	17	animal	48003		Llarena et al. (2015)
**4080-08**	45	45	Finland	2008	6	17	animal	46013		Llarena et al. (2015)
**10515-12**	45	45	Finland	2012	6	17	animal	46013		Llarena et al. (2015)
**3488-12**	45	45	Finland	2012	6	17	animal	45982		Llarena et al. (2015)
**6236-12**	45	45	Finland	2012	6	17	animal	45991		Llarena et al. (2015)
**6237-12**	45	45	Finland	2012	6	17	animal	45992		Llarena et al. (2015)
**6497-12**	45	45	Finland	2012	6	17	animal	46000		Llarena et al. (2015)
**6498-12**	45	45	Finland	2012	6	17	animal	46001		Llarena et al. (2015)
**6538-12**	45	45	Finland	2012	6	17	animal	46002		Llarena et al. (2015)
**6541-12**	45	45	Finland	2012	6	17	animal	46004		Llarena et al. (2015)
**6614-12**	45	45	Finland	2012	6	17	animal	46009		Llarena et al. (2015)
**J8**	45	45	Finland	2012	6	17	human	46021		Kovanen et al. (2014b)
**K3**	45	45	Finland	2012	6	17	human	46029		Kovanen et al. (2014b)
**OXC5171**	45	45	UK	2010	6	17	human	24772		Oxfordshire Human Surveillance Study
**OXC5225**	45	45	UK	2010	6	17	human	24811		Oxfordshire Human Surveillance Study
**OXC5231**	45	45	UK	2010	6	17	human	24817		Oxfordshire Human Surveillance Study
**OXC5236**	45	45	UK	2010	6	17	human	24819		Oxfordshire Human Surveillance Study
**OXC6259**	45	45	UK	2011	6	17	human	16056		Oxfordshire Human Surveillance Study
**OXC6278**	45	45	UK	2011	6	17	human	16075		Oxfordshire Human Surveillance Study
**OXC6313**	45	45	UK	2011	6	17	human	16110		Oxfordshire Human Surveillance Study

**OXC6339**	45	45	UK	2011	6	17	human	16136		Oxfordshire Human Surveillance Study
**OXC5850**	45	45	UK	2011	6	17	human	23667		Oxfordshire Human Surveillance Study
**OXC6894**	45	45	UK	2012	6	17	human	22089		Oxfordshire Human Surveillance Study
**OXC7014**	45	45	UK	2012	6	17	human	22201		Oxfordshire Human Surveillance Study
**OXC7018**	45	45	UK	2012	6	17	human	22205		Oxfordshire Human Surveillance Study
**OXC7025**	45	45	UK	2012	6	17	human	22212		Oxfordshire Human Surveillance Study
**OXC7074**	45	45	UK	2012	6	17	human	22258		Oxfordshire Human Surveillance Study
**OXC7090**	45	45	UK	2012	6	17	human	22272		Oxfordshire Human Surveillance Study
**OXC7099**	45	45	UK	2012	6	17	human	22281		Oxfordshire Human Surveillance Study
**OXC7315**	45	45	UK	2012	6	17	human	22356		Oxfordshire Human Surveillance Study
**OXC7634**	45	45	UK	2013	6	17	human	24563		Oxfordshire Human Surveillance Study
**OXC7766**	45	45	UK	2013	6	17	human	24575		Oxfordshire Human Surveillance Study
**OXC7776**	45	45	UK	2013	6	17	human	24583		Oxfordshire Human Surveillance Study
**OXC7911**	45	45	UK	2013	6	17	human	25041		Oxfordshire Human Surveillance Study
**OXC8007**	45	45	UK	2013	6	17	human	25491		Oxfordshire Human Surveillance Study
**OXC8195**	45	45	UK	2013	6	17		27900		Oxfordshire Human Surveillance Study
**OXC8362**	45	45	UK	2013	6	17		28050		Oxfordshire Human Surveillance Study
**OXC7225**	45	45	UK	UNK	6	17	human	22716		Oxfordshire Human Surveillance Study
**OXC7239**	45	45	UK	UNK	6	17	human	22729		Oxfordshire Human Surveillance Study
**Dg273**	45	45	UK	UNK	6	17	animal	26038		Pearson et al. (2015)
**6098-04**	7008	45	Finland	2004	6	17	animal	48008		Llarena et al. (2015)

**FI44**	45	45	Lithuania	2012	6	18	animal	46136	PRJEB15115	This study
**LS37125S**	45	45	Finland	2004	6	19	animal	46112	PRJEB15115	This study
**K2**	45	45	Finland	2012	6	19	human	46028		Kovanen et al. (2014b)
**K5**	45	45	Finland	2012	6	19	human	46031		Kovanen et al. (2014b)
**UoH540**	45	45	Finland	2001	6	17	human	2695		Zhang et al. (2015)
**71712-Vihti**	45	45	Finland	2001	6	17	human	48018		Zhang et al. (2015)
**2719-04**	45	45	Finland	2004	6	17	animal	47998		Llarena et al. (2015)
**3452-04**	45	45	Finland	2004	6	17	animal	48025		Llarena et al. (2015)
**5280-04**	45	45	Finland	2004	6	17	animal	48020		Llarena et al. (2015)
**6288-04**	45	45	Finland	2004	6	17	animal	48015		Llarena et al. (2015)
**6934-04**	45	45	Finland	2004	6	17	animal	48029		Llarena et al. (2015)
**3960-08**	45	45	Finland	2008	6	17	animal	46012		Llarena et al. (2015)
**10031-12**	45	45	Finland	2012	6	17	animal	46012		Llarena et al. (2015)
**1994-12**	45	45	Finland	2012	6	17	animal	45980		Llarena et al. (2015)
**1995-12**	45	45	Finland	2012	6	17	animal	45981		Llarena et al. (2015)
**6190-12**	45	45	Finland	2012	6	17	animal	45990		Llarena et al. (2015)
**6241-12**	45	45	Finland	2012	6	17	animal	45993		Llarena et al. (2015)
**6242-12**	45	45	Finland	2012	6	17	animal	45994		Llarena et al. (2015)
**6540-12**	45	45	Finland	2012	6	17	animal	46003		Llarena et al. (2015)
**6543-12**	45	45	Finland	2012	6	17	animal	46005		Llarena et al. (2015)
**9814-12**	45	45	Finland	2012	6	17	animal	46011		Llarena et al. (2015)
**OXC7369**	45	45	UK	2012	6	17	human	23907		Oxfordshire Human Surveillance Study
**OXC7659**	45	45	UK	2012	6	17	human	24914		Oxfordshire Human Surveillance Study
**OXC7922**	45	45	UK	2013	6	17	human	25051		Oxfordshire Human Surveillance Study
**OXC7923**	45	45	UK	2013	6	17	human	25052		Oxfordshire Human Surveillance Study
**OXC7972**	45	45	UK	2013	6	17	human	25387		Oxfordshire Human Surveillance Study
**Dg292**	45	45	UK	UNK	6	17	animal	25964		Pearson et al. (2015)

**Dg200**	45	45	UK	UNK	6	17	animal	25985		Pearson et al. (2015)
**Dg206**	45	45	UK	UNK	6	17	animal	26018		Pearson et al. (2015)
**Dg202**	45	45	UK	UNK	6	17	animal	26026		Pearson et al. (2015)
**Dg383**	45	45	UK	UNK	6	17	animal	26062		Pearson et al. (2015)
**VJA6**	45	45	Finland	2012	6	19	environment	46039		Kovanen et al. (2016)
**OXC6803**	45	45	UK	2012	7	24	human	18360		Oxfordshire Human Surveillance Study
**3458-04**	45	45	Finland	2004	7	21	animal	48009		Llarena et al. (2015)
**5095-04**	45	45	Finland	2004	7	21	animal			Llarena et al. (2015)
**5096-04**	45	45	Finland	2004	7	21	animal	48028		Llarena et al. (2015)
**OXC5192**	45	45	UK	2010	7	21	human	24787		Oxfordshire Human Surveillance Study
**OXC7103**	45	45	UK	2012	7	21	human	21185		Oxfordshire Human Surveillance Study
**OXC7326**	45	45	UK	2012	7	21	human	22365		Oxfordshire Human Surveillance Study
**OXC8026**	45	45	UK	2013	7	21	human	25589		Oxfordshire Human Surveillance Study
**OXC8336**	45	45	UK	2013	7	21		28027		Oxfordshire Human Surveillance Study
**OXC4736**	1326	45	UK	2009	7	21	human	18439		Oxfordshire Human Surveillance Study
**Dg283**	1326	45	UK	UNK	7	21	animal	25960		Pearson et al. (2015)
**3976-04**	45	45	Finland	2004	7	20	animal	48007		Llarena et al. (2015)
**5440-04**	45	45	Finland	2004	7	20	animal	47997		Llarena et al. (2015)
**OXC5332**	45	45	UK	2010	7	20	human	21316		Oxfordshire Human Surveillance Study
**OXC6419**	45	45	UK	2011	7	20	human	16214		Oxfordshire Human Surveillance Study
**OXC6624**	45	45	UK	2011	7	20	human	16373		Oxfordshire Human Surveillance Study
**OXC6916**	45	45	UK	2012	7	20	human	22111		Oxfordshire Human Surveillance Study
**OXC7401**	45	45	UK	2012	7	20	human	23937		Oxfordshire Human Surveillance Study
**OXC7813**	45	45	UK	2013	7	20	human	24611		Oxfordshire Human Surveillance Study

**OXC7235**	45	45	UK	UNK	7	20	human	22725		Oxfordshire Human Surveillance Study
**3315-08**	45	45	Finland	2008	7	22	animal	48004		Llarena et al. (2015)
**OXC7275**	45	45	UK	2012	7	22	human	22316		Oxfordshire Human Surveillance Study
**OXC7309**	45	45	UK	2012	7	22	human	22350		Oxfordshire Human Surveillance Study
**OXC7340**	45	45	UK	2012	7	22	human	22377		Oxfordshire Human Surveillance Study
**OXC7179**	45	45	UK	2012	7	22	human	22670		Oxfordshire Human Surveillance Study
**OXC8267**	45	45	UK	2013	7	22		27961		Oxfordshire Human Surveillance Study
**OXC7975**	1701	45	UK	2013	7	22	human	25390		Oxfordshire Human Surveillance Study
**OXC7987**	2197	45	UK	2013	7	22	human	25399		Oxfordshire Human Surveillance Study
**OXC6895**	45	45	UK	2012	7	21	human	22090		Oxfordshire Human Surveillance Study
**OXC6896**	45	45	UK	2012	7	21	human	22091		Oxfordshire Human Surveillance Study
**OXC7303**	45	45	UK	2012	7	21	human	22344		Oxfordshire Human Surveillance Study
**OXC7185**	45	45	UK	2012	7	21	human	22676		Oxfordshire Human Surveillance Study
**OXC7342**	45	45	UK	2012	7	21	human	23883		Oxfordshire Human Surveillance Study
**OXC7496**	45	45	UK	2012	7	21	human	24024		Oxfordshire Human Surveillance Study
**OXC7167**	6886	45	UK	2012	7	21	human	21248		Oxfordshire Human Surveillance Study
**OXC7102**	25	45	UK	2012	7	23	human	21184		Oxfordshire Human Surveillance Study
**OXC5941**	45	45	UK	2011	7	23	human	23683		Oxfordshire Human Surveillance Study
**OXC6358**	233	45	UK	2011	7	23	human	16155		Oxfordshire Human Surveillance Study
**OXC6614**	233	45	UK	2011	7	23	human	16364		Oxfordshire Human Surveillance Study

**OXC6515**	845	45	UK	2011	7	23	human	16307		Oxfordshire Human Surveillance Study
**OXC6293**	45	45	UK	2011	7	24	human	16090		Oxfordshire Human Surveillance Study
**OXC6365**	45	45	UK	2011	7	24	human	16148		Oxfordshire Human Surveillance Study
**OXC6365**	45	45	UK	2011	7	24	human	16162		Oxfordshire Human Surveillance Study
**OXC8207**	45	45	UK	2013	7	24		27912		Oxfordshire Human Surveillance Study
**OXC8213**	45	45	UK	2013	7	24		27916		Oxfordshire Human Surveillance Study
**Dg44a**	7259	45	UK	UNK	8	25	animal	25950		Pearson et al. (2015)
**Dg224**	7259	45	UK	UNK	8	25	animal	25989		Pearson et al. (2015)
**Dg307**	7259	45	UK	UNK	8	25	animal	25990		Pearson et al. (2015)
**Dg304**	7259	45	UK	UNK	8	25	animal	25991		Pearson et al. (2015)
**Dg128**	7259	45	UK	UNK	8	25	animal	26007		Pearson et al. (2015)
**Dg275**	7259	45	UK	UNK	8	25	animal	26058		Pearson et al. (2015)
**Dg117**	7259	45	UK	UNK	8	25	animal	26064		Pearson et al. (2015)
**Dg57a**	7259	45	UK	UNK	8	26	animal	25947		Pearson et al. (2015)
**Dg109**	7259	45	UK	UNK	8	26	animal	26019		Pearson et al. (2015)
**Dg104**	7259	45	UK	UNK	8	26	animal	26043		Pearson et al. (2015)
**Dg131**	7259	45	UK	UNK	8	27	animal	25988		Pearson et al. (2015)
**Dg130**	7259	45	UK	UNK	8	27	animal	26015		Pearson et al. (2015)
**OXC4721**	137	45	UK	2009	9	28	human	18430		Oxfordshire Human Surveillance Study
**OXC5331**	137	45	UK	2010	9	28	human	21315		Oxfordshire Human Surveillance Study
**OXC5434**	137	45	UK	2010	9	28	human	21380		Oxfordshire Human Surveillance Study
**OXC5744**	137	45	UK	2010	9	28	human	21471		Oxfordshire Human Surveillance Study
**OXC4907**	137	45	UK	2010	9	28	human	24467		Oxfordshire Human Surveillance Study
**OXC5318**	137	45	UK	2010	9	28		25100		Oxfordshire Human Surveillance Study

**OXC6448**	137	45	UK	2011	9	28	human	16241		Oxfordshire Human Surveillance Study
**OXC7067**	137	45	UK	2012	9	28	human	22251		Oxfordshire Human Surveillance Study
**OXC7249**	137	45	UK	2012	9	28	human	22291		Oxfordshire Human Surveillance Study
**OXC7281**	137	45	UK	2012	9	28	human	22322		Oxfordshire Human Surveillance Study
**OXC7283**	137	45	UK	2012	9	28	human	22324		Oxfordshire Human Surveillance Study
**OXC7170**	137	45	UK	2012	9	28	human	22661		Oxfordshire Human Surveillance Study
**OXC7425**	137	45	UK	2012	9	28	human	23959		Oxfordshire Human Surveillance Study
**OXC7786**	137	45	UK	2013	9	28	human	24590		Oxfordshire Human Surveillance Study
**OXC7979**	137	45	UK	2013	9	28	human	25394		Oxfordshire Human Surveillance Study
**OXC8118**	137	45	UK	2013	9	28	human	25433		Oxfordshire Human Surveillance Study
**OXC8016**	137	45	UK	2013	9	28	human	25493		Oxfordshire Human Surveillance Study
**OXC8246**	137	45	UK	2013	9	28		27945		Oxfordshire Human Surveillance Study
**OXC5208**	6017	45	UK	2010	9	28	human	24798		Oxfordshire Human Surveillance Study
**NC-017280**	137	45	UK	2000	9	30	human	GCA_000148705.1 (GenBank)		Friis et al (2010)
**OXC7881**	137	45	UK	2013	9	32	human	25014		Oxfordshire Human Surveillance Study
**OXC5343**	2109	45	UK	2010	9	31	human	21325		Oxfordshire Human Surveillance Study
**OXC6938**	2109	45	UK	2012	9	31	human	21112		Oxfordshire Human Surveillance Study
**OXC7371**	2109	45	UK	2012	9	31	human	23909		Oxfordshire Human Surveillance Study
**OXC7376**	2109	45	UK	2012	9	31	human	23914		Oxfordshire Human Surveillance Study
**OXC7377**	2109	45	UK	2012	9	31	human	23915		Oxfordshire Human Surveillance Study

**OXC8372**	2109	45	UK	2013	9	31		27872		Oxfordshire Human Surveillance Study
**OXC8375**	2109	45	UK	2013	9	31		27875		Oxfordshire Human Surveillance Study
**OXC6592**	137	45	UK	2011	9	29	human	16343		Oxfordshire Human Surveillance Study
**Dg374**	137	45	UK	UNK	9	29	animal	26029		Pearson et al. (2015)
**Dg379**	137	45	UK	UNK	9	29	animal	26037		Pearson et al. (2015)
**Dg382**	137	45	UK	UNK	9	29	animal	26055		Pearson et al. (2015)
**OXC7113**	538	45	UK	2012	9	29	human	21195		Oxfordshire Human Surveillance Study
**OXC7331**	137	45	UK	2012	9	29	human	22369		Oxfordshire Human Surveillance Study
**OXC7780**	137	45	UK	2013	9	29	human	24587		Oxfordshire Human Surveillance Study
**OXC7782**	137	45	UK	2013	9	29	human	24589		Oxfordshire Human Surveillance Study
**OXC8100**	137	45	UK	2013	9	29	human	25528		Oxfordshire Human Surveillance Study
**OXC7752**	538	45	UK	2013	9	29	human	24986		Oxfordshire Human Surveillance Study
**49-1S**	45	45	Finland	2003	9	32	animal	46113	PRJEB15115	This study
**49-15S**	45	45	Finland	2004	9	32	animal	46114	PRJEB15115	This study
**OXC7169**	45	45	UK	2012	9	32	human	21250		Oxfordshire Human Surveillance Study
**OXC8170**	45	45	UK	2013	9	32	human	25547		Oxfordshire Human Surveillance Study
**2176-08**	11	45	Finland	2008	10	33	animal	48026		Llarena et al. (2015)
**3217-08**	11	45	Finland	2008	10	33	animal	47995		Llarena et al. (2015)
**6539-12**	11	45	Finland	2012	10	33	animal	45968		Llarena et al. (2015)
**6542-12**	11	45	Finland	2012	10	33	animal	6542		Llarena et al. (2015)
**6613-12**	11	45	Finland	2012	10	33	animal	45970		Llarena et al. (2015)
**6673-12**	11	45	Finland	2012	10	33	animal	45971		Llarena et al. (2015)
**6675-12**	11	45	Finland	2012	10	33	animal	45972		Llarena et al. (2015)
**6677-12**	11	45	Finland	2012	10	33	animal	45973		Llarena et al. (2015)
**6678-12**	11	45	Finland	2012	10	33	animal	45974		Llarena et al. (2015)

**6679-12**	11	45	Finland	2012	10	33	animal	45975		Llarena et al. (2015)
**6680-12**	11	45	Finland	2012	10	33	animal	45976		Llarena et al. (2015)
**6970-12**	11	45	Finland	2012	10	33	animal	45977		Llarena et al. (2015)
**6971-12**	11	45	Finland	2012	10	33	animal	45978		Llarena et al. (2015)
**J1**	11	45	Finland	2012	10	33	human	45979		Kovanen et al. (2014b)
**FB6371**	11	45	Finland	1999	10	33	animal	46115	PRJEB15115	This study
**OXC7093**	11	45	UK	2012	10	33	human	22275		Oxfordshire Human Surveillance Study
**OXC8236**	11	45	UK	2013	10	33		27938		Oxfordshire Human Surveillance Study
**2537-08**	2219	45	Finland	2008	10	33	animal	47993		Llarena et al. (2015)
**OXC7026**	2219	45	UK	2012	10	33	human	22213		Oxfordshire Human Surveillance Study
**FI27**	11	45	Estonia	2012	10	34	animal	46134	PRJEB15115	This study
**OXC5164**	45	45	UK	2010	10	34	human	24766		Oxfordshire Human Surveillance Study
**OXC6314**	45	45	UK	2011	10	34	human	16111		Oxfordshire Human Surveillance Study
**OXC7812**	45	45	UK	2013	10	34	human	24610		Oxfordshire Human Surveillance Study
**FI17**	11	45	Estonia	2012	10	35	animal	46133	PRJEB15115	This study
**5288-04**	45	45	Finland	2004	10	34	animal	47994		Llarena et al. (2015)
**OXC5090**	45	45	UK	2010	10	34	human	24731		Oxfordshire Human Surveillance Study
**OXC5816**	45	45	UK	2011	10	34	human	23564		Oxfordshire Human Surveillance Study
**OXC7788**	45	45	UK	2013	10	34	human	24591		Oxfordshire Human Surveillance Study
**OXC5330**	1003	45	UK	2010	11	36	human	21314		Oxfordshire Human Surveillance Study
**4381-04**	1003	45	Finland	2004	11	37	animal	48022		Llarena et al. (2015)
**4384-04**	1003	45	Finland	2004	11	37	animal	46116	PRJEB15115	This study
**4385-04**	1003	45	Finland	2004	11	37	animal	46117	PRJEB15115	This study
**Dg345**	7256	45	UK	UNK	12	38	animal	26009		Pearson et al. (2015)
**Dg294**	7256	45	UK	UNK	12	38	animal	26022		Pearson et al. (2015)

Dataset two										
**CA1**	45	45	Canada	2005			animal			
**CA2**	45	45	Canada	2006			human			
**CA3**	45	45	Canada	2006			human			
**CA4**	45	45	Canada	2006			animal			
**CA6**	45	45	Canada	2007			human			
**CA7**	45	45	Canada	2007			human			
**CA8**	45	45	Canada	2007			human			
**CA9**	45	45	Canada	2007			human			
**CA11**	45	45	Canada	2007			animal			
**CA12**	45	45	Canada	2007			animal			
**CA13**	45	45	Canada	2007			animal			
**CA14**	45	45	Canada	2008			human			
**CA15**	45	45	Canada	2008			human			
**CA16**	45	45	Canada	2008			human			
**CA17**	45	45	Canada	2008			human			
**CA18**	45	45	Canada	2008			human			
**CA19**	45	45	Canada	2008			human			
**CA20**	45	45	Canada	2008			human			
**CA21**	45	45	Canada	2008			human			
**CA22**	45	45	Canada	2008			human			
**CA23**	45	45	Canada	2008			human			
**CA24**	45	45	Canada	2008			human			
**CA25**	45	45	Canada	2008			human			
**CA26**	45	45	Canada	2008			human			
**CA27**	45	45	Canada	2008			human			
**CA28**	45	45	Canada	2008			human			
**CA29**	45	45	Canada	2008			human			
**CA30**	45	45	Canada	2008			human			

**CA31**	45	45	Canada				human			
**CA32**	45	45	Canada	2011			animal			
**CA33**	45	45	Canada	2011			animal			
**CA34***	45	45	Canada	2011	4[#]		animal	46543	PRJEB15115	This study
**CA35***	45	45	Canada	2011	4[#]		animal	46544	PRJEB15115	This study
**CA36**	45	45	Canada	2011			animal			
**CA37**	45	45	Canada	2011			animal			
**CA38**	45	45	Canada	2011			animal			
**CA39**	45	45	Canada	2011			animal			
**CA40**	45	45	Canada	2011			animal			
**CA41***	45	45	Canada	2011	4[#]		animal	46542	PRJEB15115	This study
**CA42**	45	45	Canada	2011			animal			
**CA43**	45	45	Canada	2010			animal			
**CA44**	45	45	Canada	2010			animal			
**CA47**	45	45	Canada	2011			animal			
**CA48**	45	45	Canada	2011			animal			
**CA49**	45	45	Canada	2011			animal			
**CA50**	45	45	Canada	2011			animal			
**CA51**	45	45	Canada	2011			animal			
**CA53**	45	45	Canada	2011			animal			
**CA54**	45	45	Canada	2011			animal			
**CA56**	45	45	Canada	2005			human			
**CA57**	45	45	Canada	2005			human			
**CA58**	45	45	Canada	2005			human			
**CA61**	45	45	Canada	2005			human			
**CA62**	45	45	Canada	2005			human			
**CA63**	45	45	Canada	2005			human			
**CA64**	45	45	Canada	2005			human			
**CA66**	45	45	Canada	2010			animal			

**CA67**	45	45	Canada	2004			animal			
**CA68**	45	45	Canada	2004			animal			
**CA69**	45	45	Canada	2004			animal			
**CA71**	45	45	Canada	2004			environment			
**CA72**	45	45	Canada	2005			animal			
**CA73**	45	45	Canada	2005			animal			
**CA74**	45	45	Canada	2005			animal			
**CA75**	45	45	Canada	2005			animal			
**CA76**	45	45	Canada	2005			animal			
**CA77**	45	45	Canada	2006			environment			
**CA78**	45	45	Canada	2006			animal			
**CA79**	45	45	Canada	2006			animal			
**CA80**	45	45	Canada	2006			animal			
**CA81**	45	45	Canada	2006			animal			
**CA82**	45	45	Canada	2006			animal			
**CA83**	45	45	Canada	2006			animal			
**CA84**	45	45	Canada	2006			environment			
**CA86**	45	45	Canada	2006			environment			
**CA87**	45	45	Canada	2007			environment			
**CA88**	45	45	Canada	2009			environment			
**CA89**	45	45	Canada	2010			environment			
**CA90**	45	45	Canada	2010			environment			
**CA91**	45	45	Canada	2011			environment			
**CA95**	45	45	Canada	2011			environment			
**CA97**	45	45	Canada	2008			environment			
**2015TE25798***	45	45	Italy	2015			animal	46124	PRJEB15115	This study
**2015TE25090***	45	45	Italy	2015	4[#]		animal	46125	PRJEB15115	This study
**2015TE19794***	45	45	Italy	2015	6[#]		animal	46126	PRJEB15115	This study
**2015TE18310***	45	45	Italy	2015	6[#]		animal	46127	PRJEB15115	This study

**2015TE18182***	45	45	Italy	2015			animal	46128	PRJEB15115	This study
**2015TE18181***	45	45	Italy	2015			animal	46129	PRJEB15115	This study
**2015TE18180***	45	45	Italy	2015			animal	46130	PRJEB15115	This study
**2015TE18178***	45	45	Italy	2015			animal	46131	PRJEB15115	This study
**24/h/11*/****	45	45	Finland	2011			animal	46118	PRJEB15115	This study
**38/h/11*/****	45	45	Finland	2011			animal	46119	PRJEB15115	This study
**37/H/11*/****	45	45	Finland	2011			animal	46120	PRJEB15115	This study
**KAI45/12*/****	45	45	Finland	2012	4[#]		animal	46121	PRJEB15115	This study
**KAI44/12*/****	45	45	Finland	2012	4[#]		animal	46122	PRJEB15115	This study
**TOK35/12*/****	45	45	Finland	2012	6[#]		animal	46123	PRJEB15115	This study

Pearson, B.M., Louwen, R., van Baarlen, P., & van Vliet, A.H. (2015) Differential Distribution of Type II CRISPR-Cas Systems in Agricultural and Nonagricultural *Campylobacter coli* and *Campylobacter jejuni* Isolates Correlates with Lack of Shared Environments. Genome Biol Evol 7, 2663-2679

Friis,C., Wassenaar,T.M., Javed,M.A., Snipen,L., Lagesen,K., Hallin,P.F., Newell,D.G., Toszeghy,M., Ridley,A., Manning,G. & Ussery,D.W. (2010) Genomic Characterization of *Campylobacter jejuni* Strain M1. PLoS ONE 5, e12253

Kovanen, S., Kivistö, R., Rossi, M., Schott, T., Karkkainen, U.M., Tuuminen, T. et al. (2014b). Multilocus sequence typing (MLST) and whole-genome MLST of *Campylobacter jejuni* isolates from human infections in three districts during a seasonal peak in Finland. J Clin Microbiol, 52:4147-4154.

Kovanen, S., Kivistö, R., Llarena, A.K., Zhang, J., Kärkkäinen, U., Tuuminen, T. et al. (2016). Tracing isolates from domestic human *Campylobacter jejuni* infections to animal slaughter batches and swimming water using whole-genome multilocus sequence typing. Int J Food Microbiol. 2016; e-pub ahead of print 20 Mar 2016, doi: 10.1016/j.ijfoodmicro.2016.03.009

Llarena, A.K., Huneau, A., Hakkinen, M., Hänninen, M.L. (2015). Predominant *Campylobacter jejuni* sequence types persist in Finnish chicken production. PloS one, 10 10(2):e0116585. doi: 10.1371/journal.pone.0116585.ena

Revez, J., Llarena, A.K., Schott, T., Kuusi, M., Hakkinen, M., Kivistö, R., et al. (2014). Genome analysis of *Campylobacter jejuni* strains isolated from a waterborne outbreak. BMC genomics, 15 doi: 10.1186/1471-2164-15-768.

Zhang, J., Halkilahti, J., Hänninen, M.L., Rossi, M. (2015). Refinement of whole-genome multilocus sequence typing analysis by addressing gene paralogy. J Clin Microbiol 53:1765-1767.

* Genomes included in Dataset two

** Genomes of migrating wild bird isolates

[#] BAPS group level 1 with which these isolated clustered together with in the Parsnp analysis

Supplementary Table S1: Overview over the 437 *C. jejuni* genomes used in this study. The assembled genomes and raw reads of the isolates sequenced in this study were deposited to the PubMLST database (http://www.PubMLST.org/) under their respective id-numbers and under the ENA project number PRJEB15115.

Clade	Group[#]	hierBAPS L1 (L2*)	ST[##]
a	1	12	7256
	2	1(3*)	334[&&], 4791[&&]
	3	1, 8	7259[&]
b	1	2, 9	137, 230[&&], *538,* 583, 2109[&&], *6017*
	2	9(32*)	45[&],,137
	3	10	11, 45[&], *2219*
	4	4	45[&]
	5	7(22*)	45[&], *1701, 2197*
	6	5, 6, 11, 7	25 , 45[&], 233, 845, 1003, 1326, 6886, 7008

[#] Group defined with bootstrap supporting value ≥ 80% as showed in figure S1A

[##] Singletons are marked in italics

[&] STs divided between different BAPS groups on L1

[&&] Monophyletic STs forming ST-specific populations on L1 and L2

Supplementary Table S2 Overview of cladogistic relationship between STs and population structure. BAPS level two is indicated by an asterix.

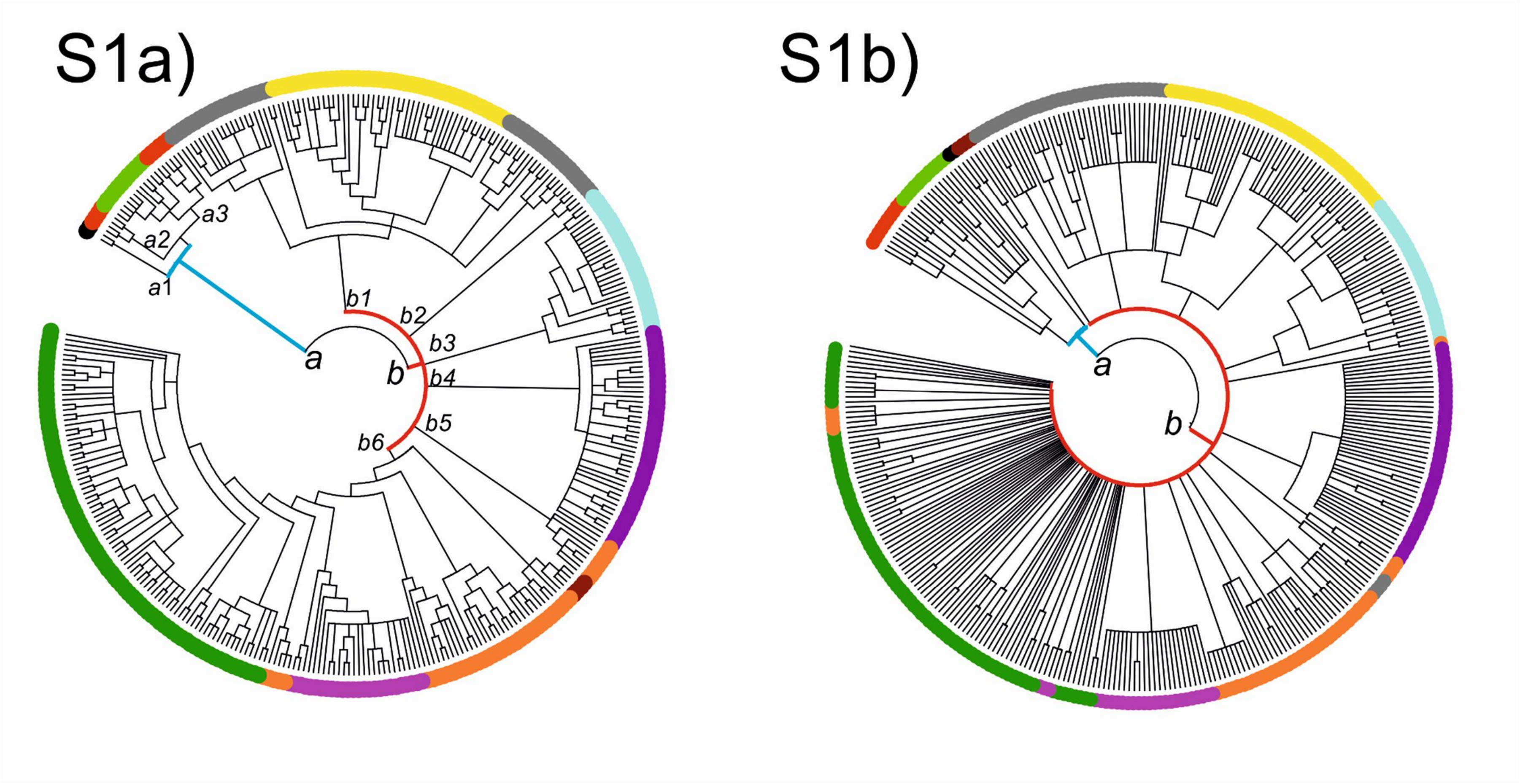

Supplementary Figure S1 Cladogram of core (S1a) and accessory (S1b) genome using >80% bootstrap values. Clades *a* and *b* are marked as red and blue branches respectively and BAPS level 1 clustering is depicted with colors on the outer ring: black; BAPS 12 red; BAPS 1 light green;8 grey; BAPS 9 yellow; BAPS 2 turquoise; BAPS 10 purple; BAPS 4 orange; BAPS 7 brick red; BAPS 11 pink; BAPS 5 dark green; BAPS 6. A) The clade numbers are indicated with letter *a* or *b* designating clade and numbers as close to the divergent node as possible. Two of the six monophyletic groups on the *b*-clade (group b1 and b6) consisted of several BAPS populations and while group b1 contained strains from multiple STs (ST-230 ST-583 ST-137 ST-2109) group b6 contained solely ST-45 strains belonging to BAPS 5 6 or 7 excluding singleton STs (i.e. STs represented by {less than or equal to} two isolates). The singletons were scattered through the ML tree with the exception of ST-7256

which formed its own BAPS population 12. B) Inclusion of the accessory genome disrupted the clonalframe of clade b resulting in star-shaped evolutionary relationship.
